# Improving the Quality of Adult Mortality Data Collected in Demographic Surveys: Validation Study of a New Siblings' Survival Questionnaire in Niakhar, Senegal

**DOI:** 10.1371/journal.pmed.1001652

**Published:** 2014-05-27

**Authors:** Stéphane Helleringer, Gilles Pison, Bruno Masquelier, Almamy Malick Kanté, Laetitia Douillot, Géraldine Duthé, Cheikh Sokhna, Valérie Delaunay

**Affiliations:** 1 Heilbrunn Department of Population and Family Health, Mailman School of Public Health, Columbia University, New York, New York, United States of America; 2 Research Unit “Mortality, Health, Epidemiology,” French National Institute for Demographic Studies (INED), Paris, France; 3 Université Catholique de Louvain-la-Neuve, Louvain-La-Neuve, Belgium; 4 Ifakara Health Institute, Dar-es-Salaam, Tanzania; 5 URMITE, UMR CNRS 6236, IRD 198, Aix Marseille Université, Marseille, France; 6 Joint Campus UCAD-IRD of Hann, BP 1386 CP 18524, Dakar, Senegal; 7 LPED, UMR151, Institut de Recherche pour le Développement, Aix-Marseille Université, Marseille, France; Columbia University Mailman School of Public Health, United States of America

## Abstract

**Background:**

In countries with limited vital registration, adult mortality is frequently estimated using siblings' survival histories (SSHs) collected during Demographic and Health Surveys (DHS). These data are affected by reporting errors. We developed a new SSH questionnaire, the siblings' survival calendar (SSC). It incorporates supplementary interviewing techniques to limit omissions of siblings and uses an event history calendar to improve reports of dates and ages. We hypothesized that the SSC would improve the quality of adult mortality data.

**Methods and Findings:**

We conducted a retrospective validation study among the population of the Niakhar Health and Demographic Surveillance System in Senegal. We randomly assigned men and women aged 15–59 y to an interview with either the DHS questionnaire or the SSC. We compared SSHs collected in each group to prospective data on adult mortality collected in Niakhar. The SSC reduced respondents' tendency to round reports of dates and ages to the nearest multiple of five or ten (“heaping”). The SSC also had higher sensitivity in recording adult female deaths: among respondents whose sister(s) had died at an adult age in the past 15 y, 89.6% reported an adult female death during SSC interviews versus 75.6% in DHS interviews (*p* = 0.027). The specificity of the SSC was similar to that of the DHS questionnaire, i.e., it did not increase the number of false reports of deaths. However, the SSC did not improve the reporting of adult deaths among the brothers of respondents. Study limitations include sample selectivity, limited external validity, and multiple testing.

**Conclusions:**

The SSC has the potential to collect more accurate SSHs than the questionnaire used in DHS. Further research is needed to assess the effects of the SSC on estimates of adult mortality rates. Additional validation studies should be conducted in different social and epidemiological settings.

**Trial Registration:**

Controlled-Trials.com ISRCTN06849961

*Please see later in the article for the Editors' Summary*

## Abstract 2

Stéphane Helleringer and colleagues conducted a validation study in Niakhar, Senegal to investigate whether a new approach, sibling survival calendars, improves the quality of adult mortality data collected in demographic surveys.

*Please see later in the article for the Editors' Summary*

## Editors' Summary

**Background:**

Governments and international health agencies need accurate information on births and deaths in populations to help them plan health care policies and monitor the effectiveness of public health programs. The most common way of collecting information on vital statistics in an area or country is through civil registration, an administrative system used by governments to record vital events that occur in their populations. In low-to-middle-income countries that have limited resources to devote to such a system, unconventional techniques are often used to estimate mortality levels and trends. One such method is siblings' survival histories collected while conducting a health or other type of public survey. Those surveyed are asked to list all their maternal siblings by birth order and report survival status and current age (for living siblings) or age at death (for deceased siblings).

Use of siblings' survival histories leaves the accuracy of the mortality record vulnerable to reporting errors and selection bias. Selection bias is a statistical prejudice that is introduced by the choice of the individuals or groups taking part in the analysis. Reporting errors occur when an individual fails to report a sibling's death, misreports the age of a sibling, or does not recall the exact date when a sibling died.

**Why Was This Study Done?:**

This study was conducted to estimate whether modifying a standard siblings' survival history questionnaire could improve the accuracy of data obtained. The researchers conducted a study in Niakhar, Senegal, using a modified siblings' survival history questionnaire to incorporate some innovative techniques for assisting memory recall, such as recall cues, to help prevent omissions. The researchers also introduced an event history calendar format to help with more accurate reporting of dates. This modified questionnaire is called the siblings' survival calendar.

**What Did the Researchers Do and Find?:**

The researchers randomly assigned men and women registered by the Niakhar Health and Demographic Surveillance System, aged 15–59 years old, to an interview with the basic questionnaire or the modified siblings' survival calendar. The modifications included emphasizing the importance of accurate recall before the participant started and asking respondents to list their maternal siblings in the order that they came to the mind instead of birth order. Also, the researchers used supplementary interviewing techniques designed to stimulate the recall of potentially omitted siblings and also used an event history calendar approach for collecting data on ages at, and dates of, vital events that had affected the siblings of a respondent.

The researchers compared the results from the two survey instruments and precise data on adult mortality collected by continuous demographic surveillance in a small area of Senegal. They found that the calendar survey improved the sensitivity of survey data in recording adult female deaths. In addition, the modified questionnaire significantly reduced age and date heaping (the tendency of respondents to round off dates) observed with the basic questionnaire. The modified questionnaire took six minutes longer to complete on average than the basic questionnaire.

**What Do These Findings Mean?:**

These findings suggest that a new approach (the siblings' survival calendar) that uses simple interview tools to improve estimates of mortality in resource-limited countries is feasible and may improve the quality of the data from siblings' survival histories, particularly in reporting female deaths. Although the study was limited by sample selectivity, limited external validity, and multiple testing, the findings suggest that this new approach has the potential to allow the collection of more accurate data from siblings' survival histories than that collected from the current questionnaire. The next step is to validate these findings in other settings.

**Additional Information:**

Please access these websites via the online version of this summary at http://dx.doi.org/10.1371/journal.pmed.1001652.

- The World Health Organization Regional Office for Africa has a page on vital statistics
- Information on demographic data available in Senegal can be accessed on the website of the Agence Nationale de la Statistique et de la Démographie (in French)
- The UN Statistics Division has a page on their vital registration and vital statistics coverage assessment
- The World Mortality Report 2013 presents the latest mortality estimates developed by the Population Division of the United Nations, including the probability of dying between the ages of 15 and 60 years
- The Institute for Health Metrics and Evaluation has developed a visualization tool that allows one to see how siblings' survival histories are adjusted to generate estimates of adult mortality
- More information on Health and Demographic Surveys surveillance in Niakhar, Senegal, is available (in French) on the website of the Institut de Recherche pour le Développement

## Introduction

In countries with limited vital registration, demographers increasingly rely on “unconventional techniques” to estimate mortality levels and trends [1]. Such techniques utilize information collected during censuses or surveys about the survival of the close relatives of a respondent (e.g., her parents or the members of her household). Estimates derived from these unconventional techniques are replacing estimates from model life tables in major comparative mortality studies [2]–[5]. In particular, siblings' survival histories (SSHs) collected during nationally representative surveys (e.g., Demographic and Health Surveys [DHS]) are now one of the primary sources of information on adult mortality in countries with limited vital registration. In SSHs, survey respondents are asked to list all their maternal siblings by birth order and report their survival status, their age, and, if deceased, their age at, and date of, death. SSHs have been used to evaluate the mortality impact of large global health initiatives such as the United States President's Emergency Plan for AIDS Relief [6] or to estimate the number of deaths due to conflict or genocide [7]–[10]. SSHs are also increasingly used to estimate pregnancy-related mortality [11]–[13] and monitor progress towards Millennium Development Goal 5 [14].

The use of SSHs to estimate adult mortality has long been debated among demographers. There are two main concerns: SSHs may suffer from sample selection biases [15], and they may be affected by reporting errors. Sample selection biases emerge when individuals at risk of experiencing an event (e.g., death) do not all have the same probability of being included in a dataset. This occurs in SSHs because (1) individuals who died during the risk interval are necessarily excluded and (2) several siblings can potentially report on the same family. If adult mortality is associated with family size, then estimates of adult mortality rates will be biased. Analytical corrections for sample selection biases have been proposed [16],[17] and evaluated [18].

Reporting errors refer to situations where the SSH reported by a survey respondent differs from the true survival experience of his/her sibling(s). Such errors have a significant impact on estimates of adult mortality rates [19]. Demographers have primarily sought to diagnose reporting errors in SSH datasets [20]. Only recently have they tried to correct such errors through analytical procedures. In particular, researchers working on the Global Burden of Disease 2010 study proposed an innovative adjustment method, the “corrected sibling survival” (CSS) method, based on the comparison of SSH data reported in consecutive cross-sectional surveys [17]. If SSHs are collected, say, in 2005 and 2010, then adult deaths that occurred in 2004 should be reported by respondents in both the 2005 and the 2010 surveys. In pooled analyses of mortality risk, the CSS method can thus estimate how much the reporting of deaths decays with the amount of time elapsed between a death and the survey. This parameter can then be used to adjust estimates of mortality risks upwards [3],[17].

The CSS method, however, requires multiple SSH surveys and makes strong assumptions about patterns of respondent recall. For example, it assumes that the most recent deaths (i.e., deaths that occurred within 2–3 y of the survey) are completely and accurately reported during SSH surveys. However, if respondents have not had any recent contact with one of their brother(s)/sister(s) because of migration, for example, they may not be aware that s/he has recently died. Similarly, respondents may be reluctant to report recent deaths attributable to stigmatized causes (e.g., HIV/AIDS). Finally, some errors may emerge because of interviewer behavior: SSHs can be time-consuming to collect, and interviewers may be tempted to “skip over” some of the siblings of a respondent (including recently deceased ones). In a study we conducted in Bandafassi, Senegal, we found that even some deaths that occurred within 2–3 y of the survey were omitted during SSHs [11],[19]. In such instances, adjustments derived by the CSS method would likely still underestimate the extent of adult mortality.

In this study, we followed a different strategy to address reporting errors in SSHs. Instead of pursuing analytical adjustment strategies, we sought to improve the quality of SSH data. To do so, we modified the standard SSH questionnaire used during DHS by (1) including supplementary interviewing techniques (e.g., recall cues) designed to prevent omissions of siblings in SSHs and (2) adopting an event history calendar format that has been shown to improve reports of ages and dates in demographic surveys. We then conducted a validation study of this new SSH questionnaire, which we call the siblings' survival calendar (SSC), in Niakhar, Senegal. Niakhar has been the site of a Health and Demographic Surveillance System (HDSS) since 1962, which has collected prospective data on adult mortality since its launch. It thus provides a high-quality reference dataset to measure the accuracy of the SSH data. We hypothesized that the SSC would improve the quality of adult mortality data, relative to the DHS questionnaire.

## Methods

### Ethics Statement

This study was approved by the Columbia University Medical Center institutional review board (Protocol AAAI9159) and by the Ethics committee of Senegal's Ministry of Health and Social Action (SEN 12/11). Study participants provided informed consent in writing prior to participating in the study.

### Overview of Study Design

This is a retrospective validation study of SSH data collected during surveys in the lower-middle-income country of Senegal. It estimates the accuracy of two survey instruments (the SSC and the standard DHS questionnaire, both described in detail below) by comparing the SSH data they yield to a prospective dataset on adult mortality, the Niakhar HDSS (reference dataset). We first selected and enrolled a stratified sample of individuals who had ever been registered by the Niakhar HDSS. In doing so, we oversampled individuals who had experienced at least one adult death among their maternal siblings, according to the HDSS. We then randomly allocated study participants to either an interview with the DHS questionnaire (control group) or to an interview with the SSC (experimental group). Finally, we compared the quality of SSH data obtained in each study group.

### Study Setting

The study focused on the population of the Niakhar HDSS site, located 120 km southeast of Dakar, Senegal's capital. The population covered by the HDSS lives in 30 villages comprising ≈44,000 inhabitants as of 1 January 2013. Most of the population belongs to the Sereer ethnic group, with Wolof, Toucouleur, and Laobe minorities. The main language is Sereer but many people also speak Wolof, the most common language in Senegal. The main religious groups in the area are Muslim (≈80%) and Christian (≈20%). Households in Niakhar live traditionally on one food crop (millet) and one cash crop (groundnuts). They also raise a few cattle. The climate is typical of the sub-Sahel. The three largest villages in the area include health facilities, weekly markets, daily buses to Dakar, and several shops. The educational level is low: 50% of men and 75% of women in the HDSS population have never attended primary school. High levels of mobility, both permanent and temporary, also characterize the area. A large proportion of Niakhar residents move to Dakar, where they seek employment. A more detailed description of the Niakhar HDSS is given elsewhere [21].

### Measurement Tools for Adult Mortality

#### Reference dataset

Activities of the Niakhar HDSS started in 1962 in eight villages of the Niakhar area and were later expanded to 30 villages in 1983. An initial baseline census was carried out in 1962, followed by another census in 1983, when the study area was expanded. Since these censuses, data on demographic events have been collected from household informants during household visits. Study interviewers use a printed roster of household residents and inquire about the vital status of each household member, as well as possible changes in marital status and births since the previous household visit. New household members (including in-migrants) are added to the roster. Each individual who ever resided in the study area since the start of the HDSS has been assigned a unique ID number, under which HDSS data are stored. From 1962 to 1987, household visits were conducted yearly. From 1987 to 1997, household visits were conducted weekly because of requirements of vaccine trials conducted in the Niakhar area [22]–[24]. Between 1997 and 2007, household visits were conducted every 3 mo, and between 2008 and 2013, every 4 mo.

In the Niakhar HDSS dataset, the date of birth of a population member is ascertained in one of two ways. It is recorded prospectively if s/he is born in the HDSS population between two household visits. It is assessed retrospectively if s/he was already present in the population at the time of the initial household census (i.e., 1962 or 1983) or if s/he first entered the HDSS population after birth, by migration. The age of population members is thus known with varying degrees of precision and certainty depending on the way they entered the HDSS dataset.

The date of other vital events (e.g., deaths and migrations) is ascertained through household visits, when changes in household membership are recorded. The household informant is asked to report the day and month when a death/migration occurred. The dates of vital events are thus known with varying degrees of precision, depending on the frequency of household visits. For each event, the age at the time of the event is calculated as date of event minus date of birth of the individual. Migrants who move to another household of the HDSS study population are assigned a new residence and continue being part of the longitudinal follow-up under their same ID number. On the other hand, migrants who move outside of the HDSS area (e.g., Dakar) are lost to follow-up: they are not tracked, and their relatives are not asked to report their vital status. As a result, it is not known whether they are still alive at any time after their migration. If a migrant returns to the HDSS area after some time outside, s/he is reassigned his/her previous ID number so as to avoid duplication of individuals in the HDSS dataset.

The Niakhar HDSS allows researchers to identify sibships (i.e., brothers and sisters having the same biological mother) among individuals who have ever resided in the HDSS population. The identification of sibships is possible because every population member is potentially linked to his/her biological mother through a mother ID number. The mother ID number is attributed either at the time of birth (if the mother gave birth in the HDSS area) or the first time an individual enters the HDSS population (i.e., initial census or after in-migration). We used these data to identify the sibships of potential respondents and measure the rate of omissions of siblings' deaths in each study group (see below). For some members of the HDSS population, the mother ID number may be missing because their biological mother may never have been a member of the HDSS population or because the information reported during household visits was not sufficient to establish a link between mother and child. Similarly, some sibships may be only partially identified if, for example, some of the siblings were born outside of the HDSS area.

#### Control questionnaire

We used the standard DHS questionnaire (i.e., the maternal mortality module). It consists of a single name-generating question, asking respondents to list all their maternal siblings (i.e., siblings with the same biological mother), starting from the oldest. Then, for each nominated maternal sibling, respondents are asked (1) whether the sibling is male/female, (2) whether s/he is still alive, (3) if alive, how old s/he currently is, (4) if deceased, how old s/he was when s/he died, and (5) how long ago (in years) did the death occur. For female siblings deceased above age 12 y, the DHS instrument also includes questions about the circumstances of the death, e.g., whether she died during pregnancy, at the time of delivery, or within 2 mo after the end of a pregnancy or childbirth.

#### Experimental questionnaire: the siblings' survival calendar

The SSC includes four major modifications of the DHS questionnaire. First, interviewers were instructed to sensitize respondents to the issue of misreporting prior to beginning the SSH interview. To do so, interviewers used a standardized script stressing the importance of accurate recall. Second, instead of asking respondents to list their maternal siblings by birth order, the SSC asked respondents to list their maternal siblings in the order that they came to mind (“free recall”). This modification was adopted because studies in social psychology indicate that imposing sorting constraints in name-generating questions limits the number of persons reported during surveys about social relationships [25],[26].

Third, the SSC used supplementary interviewing techniques designed to stimulate the recall of potentially omitted siblings. These techniques have been used previously in sexual networks research and in retrospective studies of dietary intake [27]–[31], for example. They included nonspecific prompting, reading back the list of nominated siblings, and recall cues. Nonspecific prompting involves asking respondents whether there are other maternal siblings they may have omitted to list. Interviewers were encouraged to prompt nonspecifically after the initial list of freely recalled siblings was obtained. At that time, interviewers were also instructed to read back the list of nominated siblings to the respondent slowly, starting from the sibling nominated last. This procedure gives survey respondents an additional opportunity to recall their siblings [29].

Recall cues were designed using findings from a validation study of SSH data conducted in Bandafassi, a HDSS site in southeastern Senegal [32]. During this study, we found that maternal siblings were more likely to be omitted during SSH interviews if they were deceased, had migrated away from the community of origin, had a different biological father than the respondent, or had not co-resided with the respondent [19]. We thus developed a set of recall cues that addressed these factors. The interviewer introduced cues to the respondent one at a time, asking him/her whether s/he may have omitted one or more maternal siblings who correspond to a cue.

Finally, we adopted an event history calendar approach to collecting data on ages at, and dates of, vital events that affected the siblings of a respondent. Event history calendars are designed to help respondents accurately report the timing of past events [33]–[36]. Such calendars reduce recall error and significantly increase data reliability [36]–[41], but they have not been used in the collection of SSHs. The event history calendar we designed is a large grid (A3 paper) bracketed by years from 1953 to 2013. It is composed of three sections: a landmarks section, a respondent section, and a siblings section. Landmarks are events that are likely to be remembered by most respondents. These include, for example, major political events (e.g., independence of Senegal), natural disasters (e.g., droughts), and sporting events (e.g., Senegal reaching the quarterfinals of the 2002 World Cup). Because SSH data are most frequently collected during national surveys, we included only national events as landmarks, rather than also including more local events. For example, we did not include electrification of a respondent's village or the death of a local chief in this section, even though these are events that are likely well remembered by study respondents. The respondent section recorded events having affected the respondent him/herself in four key domains: residence, marriages, births, and schooling. The sibling section was used to (1) reorder the list of all nominated siblings by birth order and (2) elicit the date of birth—and possibly date of death—of each sibling. Events recorded in the landmarks and respondent sections aimed at anchoring the reporting of events that affected siblings. Compared to the DHS questionnaire, the structure of the SSC is more flexible and lends itself to repeated probing and crosschecking of answers. The SSC (in French) is available as Questionnaire S1. Both the SSC and the DHS questionnaire included a small number of questions on the socio-demographic characteristics of respondents (e.g., schooling, religion).

### Study Participants

Study participants were selected among individuals who had ever been registered by the Niakhar HDSS. Individuals were eligible if they were aged 15 to 59 y on 1 January 2013 according to the HDSS dataset and they had at least one known sibling in the HDSS dataset. We excluded members of the HDSS population who had no known siblings or who had a missing/invalid/inconsistent mother ID number in the HDSS dataset. The SSH data these individuals may report during a retrospective SSH survey cannot be validated against the HDSS data. Study participants included individuals who resided in the Niakhar HDSS area at the time of the survey, individuals who were temporarily absent from their residence in the HDSS area, and individuals who had permanently migrated outside of the HDSS area prior to the survey. The latter two groups were traced to their new places of residence following methods used in migration studies conducted in sub-Saharan countries (e.g., [42]).

### Participant Recruitment

Recruitment was conducted on the basis of household visits. Participants who were still resident of the Niakhar HDSS were visited up to three times by the study team. For participants who had migrated outside of the HDSS area or were temporarily absent due to seasonal migration, we first conducted a short “migration inquiry” with members of their last known HDSS household. We asked a household informant about their destination (region, city/village, neighborhood) and tried to obtain at least one contact phone number for the migrant. The form used to collect this information (in French) is shown in Questionnaire S2.

Because of resource constraints, we could not attempt to trace all absent residents and migrants selected for study participation. Some former members of the HDSS population had moved abroad (e.g., The Gambia) or to areas of Senegal that are hard to reach (e.g., Valley of the Senegal River, Casamance). We thus delineated a “tracing area” within which the study team sought to contact absent residents and migrants for enrollment. This tracing area included Dakar and its suburbs, Mbour and surrounding areas, as well as the area within an 80-km radius of Niakhar. If the migrant or absent resident was reported to reside in the tracing area, members of the study team sought to get in touch with him/her by phone and schedule an appointment to conduct the interview in person.

### Participant Sampling

#### Primary sample

**Figure 1:**
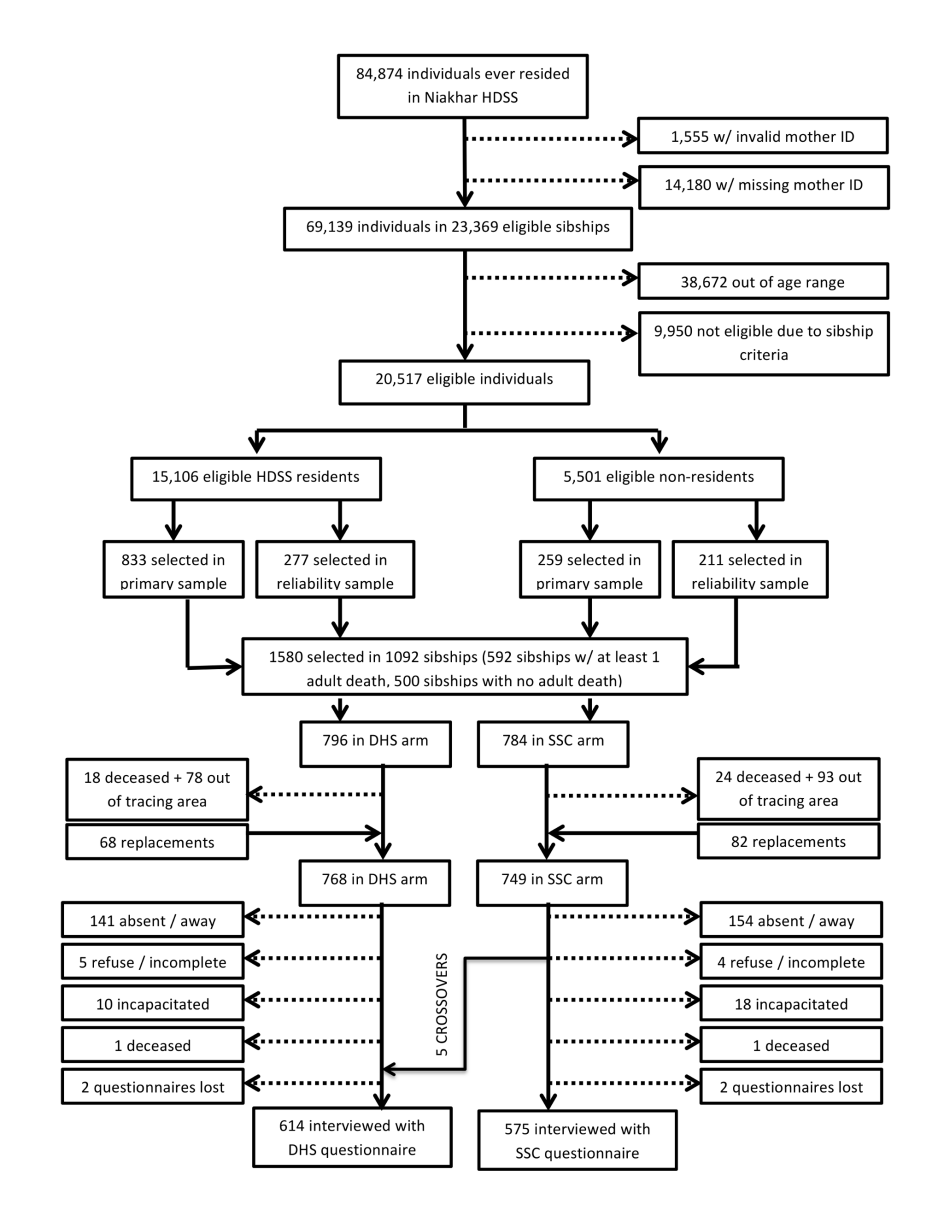
Flow diagram of the enrollment process.

The study enrollment process is summarized in Figure 1. First, we identified all individuals who met eligibility criteria among individuals ever registered by the Niakhar HDSS. The selectivity resulting from age- and sibship-related criteria is described in Table S1. Then, we identified sibships in which at least one adult death had occurred over the course of the HDSS and sibships in which there was no known adult death among known siblings. We selected all sibships with adult death(s) for inclusion (*n* = 592), then we selected an additional 500 sibships at random among sibships in which all siblings registered by the HDSS were still alive. Since adult mortality is a relatively rare event, this stratified sampling was necessary to ensure sufficient reports of adult deaths during the validation study. In total, 1,092 sibships formed our “primary sample.” Among these sibships, we selected one participant to interview at random among eligible members.

#### Reliability sample

Among the 592 sibships of the primary sample in which there was at least one adult death among siblings, we sampled an additional participant at random among the remaining eligible individuals. This additional sampling was designed to enable an assessment of the inter-sibling reliability of adult mortality data collected in each study group (“reliability sample”). In total, 488 additional participants were selected through this procedure (Figure 1). The same interviewer could not interview two individuals from the same sibship.

#### Replacement sample

Finally, we replaced sampled respondents who had migrated outside of the tracing area by interviewing another randomly selected member of their sibship. If there were no potential replacements in a respondent's sibship, the respondent was not replaced. Similarly, we replaced sampled respondents who were deceased or incapacitated by another member of their sibship. In total, 150 replacements were thus added to the study sample (Figure 1).

### Randomization

Participants selected in the primary sample were randomized 1∶1 to an interview with the DHS questionnaire (DHS group) or the SSC (experimental group). In the primary sample, randomization was stratified by gender, residence at last HDSS visit (in HDSS area or elsewhere), and composition of the respondent's sibship (at least one adult death versus no adult death among siblings). Randomization was conducted using computer-generated random number sequences in Stata 12. Participants selected as part of the reliability or replacement samples were automatically assigned to the same interview questionnaire as their sibling in the primary sample. As a result of the selection processes in the reliability and replacement samples, the final numbers of participants allocated to each study group were not perfectly equal (e.g., some respondents to be replaced did not have another eligible sibling, see Figure 1).

### Data Collection

#### Study process

In a number of methodological trials of survey questionnaires or interviewing techniques, control and experimental groups are surveyed concurrently either by the same study team or by different study teams (e.g., [37],[43],[44]). This approach to data collection raises potential contamination concerns: interviewers may be tempted to use the methodological innovations of the experimental questionnaire during control interviews. For example, they may informally incorporate some of the recall cues in control interviews. If separate study teams conduct control and experimental interviews concurrently, (1) contamination may still occur if study teams communicate with each other, and (2) interviewer effects may confound study results if teams are small (<30 interviewers per team). We decided to conduct control and experimental group interviews consecutively with the same team of interviewers. After a short training (see below), interviewers first collected the control group data using the DHS questionnaire. They were then trained in the use of the SSC. Finally, they collected the experimental group data using the SSC. This approach to data collection ensured that control interviews occurred without knowledge of the methodological innovations introduced in the SSC. All study data and outcomes were measured in one visit, there was no follow-up after the SSH interview. Neither the interviewer, nor the study participants were blinded, but interviewers did not have access to prior HDSS information about the sibship of the respondents they interviewed (e.g., whether the respondents belonged to a sibship where adult deaths had occurred).

#### Study team and training

We recruited interviewers with prior experience of DHS data collection in Senegal. We did so for two reasons: first, to ensure that the SSC would be feasible for interviewers with qualification and skills comparable to those of interviewers typically employed during DHS; second, we hypothesized that hiring interviewers with DHS experience would enhance the external validity of our validation study data. We thus contacted the Senegalese National Agency of Statistics and Demography, which implements the DHS in Senegal. We asked officers in charge to recommend ten potential interviewers who had previously worked as interviewers during one of the DHS conducted in the country. After a short selection process (based on an aptitude test and interview), we selected eight interviewers. Seven interviewers had participated in the 2010/2011 Senegal DHS, whereas one interviewer had participated only in the 2005 DHS.

All interviewers spoke Wolof, but only one interviewer was fluent in Sereer. Training for each questionnaire lasted 3 d. The study investigators (S.H., A.M.K., L.D., and B.M.) first reviewed each item on the questionnaire with the interviewers, addressing questions and discussing translations of key terms. Then interviewers worked in pairs on a series of role-playing exercises, during which they interviewed each other. Study investigators provided feedback on interviewing techniques and corrected errors. Finally, each interviewer conducted 2–4 practice interviews with inhabitants of the town of Niakhar (outside of the HDSS), who were not part of the study sample.

#### Data editing and supervision

Two of the study interviewers also served as team supervisors. They were asked to (1) track completion of the study sample, (2) check completed questionnaires for errors (e.g., missing data, incoherent ages), and (3) provide feedback to interviewers and study investigators. At least one of the study authors was present in Niakhar at all times during the course of the study. Data collected in each study group were double-entered using Epi Info.

### Analysis

The analyses we report here are exploratory, previously unplanned analyses of the validation study data. They focus on (1) metrics that are commonly used by demographers in evaluating the quality of SSH data and (2) simple sibship-level comparisons between the reference HDSS dataset and the SSH data collected using the SSC versus DHS questionnaires. We chose to conduct such analyses because they permit a direct comparison between our validation study results and data quality assessments conducted with data from national DHS [20], hence allowing an assessment of the external validity of study results. In contrast, the planned analyses of the validation study data focus on outcomes measured at the sibling level by record linkage, i.e., matching the report of a particular sibling's survival obtained through SSH to the record of that same sibling's survival in the HDSS dataset. Record linkages permit precise measurement of the proportion of siblings who are added/omitted during SSHs (planned primary outcome), as well as measurement of age and date errors (planned secondary outcomes) in reports of SSH [19]. But record linkages can rarely be implemented outside of a small number of HDSS populations because they require detailed lists of a respondent's siblings as well as sufficient identifying information about each sibling. Analyses of planned study outcomes are still ongoing and will be reported later.

#### Type of analysis

All study group comparisons reported below were conducted on an intention-to-treat basis, i.e., with participants included in their randomly assigned study group. During the course of the study, however, five participants assigned to the SSC group were interviewed with a DHS questionnaire by error (see Figure 1). We thus also conducted an as-treated analysis in which we reclassified these five participants as members of the DHS group (i.e., the questionnaire with which they were actually interviewed). The as-treated analysis yielded results similar to those of the intention-to-treat analysis.

#### Data quality assessment

We first measured the proportions of missing data on current age of live siblings and age at death and date of death of deceased siblings. We tested for differences in the extent of missing data between study groups using χ^2^ tests of the association between categorical variables. Next, we measured age/date “heaping” in SSH reports, i.e., respondents' propensity to report ages and dates ending in round numbers (e.g., multiples of five or ten). Heaping is an oft-used indicator of deficiencies in demographic data [45]–[48]. We measured age and date heaping in each study group by computing the following heaping ratio for each age/date:
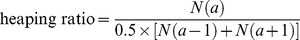
(1)where *N*(*a*) represents the number of nominated siblings at age/date *a*. This heaping ratio is an indicator of the excess number of deaths reported to have occurred at a certain date or age. For assessment of the external validity of study results, we also investigated heaping patterns in the Senegal 2010/2011 DHS.

#### Data accuracy

We tested whether the SSC was more accurate in recording adult deaths among the maternal siblings of a survey respondent than the DHS questionnaire. We measured accuracy by the concordance—at the sibship level—between SSH data reported in each study group and the Niakhar HDSS dataset. For example, let us consider a respondent who has one adult sister who died in the last 15 y according to the HDSS. The SSH reported by this respondent is said to be “concordant” if the respondent also reported an adult death of one of his sisters during that time frame. The SSH is “discordant” if the respondent did not report any adult death or reported only adult deaths among his/her brothers. On the other hand, among respondents whose known adult sisters were all still alive according to the HDSS, an SSH was deemed “concordant” if the respondent did not report any adult death among his/her sisters.

We thus defined the sensitivity of SSH data as the proportion of concordant SSHs among respondents with at least one adult death among their sisters/brothers according to the HDSS dataset. Sensitivity was measured separately by gender of the deceased. We measured the sensitivity of each questionnaire in capturing adult deaths that occurred in the 15 y prior to the survey. We selected this reference period (15 y) because this corresponds to the reference period used in analyses of the global burden of disease relying on SSH data [3].

The specificity of SSH data was defined as the proportion of concordant SSHs among respondents with no adult death among their sisters/brothers in the past 15 y according to the reference HDSS dataset. We measured the specificity of SSH data because we were concerned that the SSC may lead to false reports of deaths among siblings during SSHs. Such false reports may happen because of repeated probing and prompting (e.g., additions of cousins or other relatives of the respondents).

#### Interviewing time

We evaluated the amount of time required to complete each questionnaire (in minutes). If the SSC requires significantly more time to complete than the DHS instrument, it may be impractical to adopt in large-scale nationally representative surveys such as the DHS.

#### Statistical analyses

We tested for differences in the specificity and sensitivity of SSH data between study groups (SSC versus DHS questionnaire). To do so, we used logistic regressions controlling for stratification variables (i.e., gender of the respondent, his/her residence, and sibship composition), as recommended for analysis of stratified randomized trials [49]. Based on the observed values of sensitivity/specificity for each questionnaire, we calculated the true proportion of respondents with at least one adult death among their adult sisters/brothers. We used the formula [50]

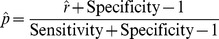
(2)where 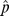 is the true proportion of respondents with at least one adult death among their sisters, and 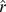 is the proportion of respondents reporting at least one adult death during their SSH interview. We let 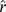 vary from 0% to 50%, and we estimated the extent of bias in estimates of 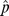 as 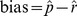. We then conducted subgroup analyses in which we tested whether the effects of the SSC on the sensitivity/specificity of SSH data varied across different respondent characteristics. These characteristics included: gender, age (<25 y old, 25–34 y old, 35–44 y old, ≥45 y old), place of interview (in Niakhar HDSS versus in the tracing area), education (no schooling versus primary schooling versus secondary schooling or higher) and religion (Muslim versus Christian). To do so, we used the Mantel-Haenszel test of the homogeneity of odds ratios across strata of a classifying variable [51], as recommended in guidelines for subgroup analyses [52],[53]. In sibships where two siblings were interviewed, we measured agreement in reported SSHs using Cohen's Kappa [54]. Finally, we compared the average duration of interviews between study groups using non-parametric tests of differences in median. All tests of statistical significance were adjusted for the clustering of respondents within sibships.

#### Sample size and power analysis

We initially planned to contact 698 respondents, but we managed to enroll close to 1,200 participants because of shorter interviewing times and higher interviewer productivity (i.e., daily number of interviews) than expected. The study was designed to measure medium effects of the SSC on the primary study outcome, i.e., the proportion of adult siblings omitted during SSH interviews assessed through record linkages (see Text S1). Since the study was not designed to measure the indicators we report here (e.g., sensitivity), we conducted tests of the equivalence of sensitivity/specificity indicators between the SSC and DHS questionnaires. Based on the available sample size, this approach allows identifying an equivalence interval (p−Δ; p+Δ) that likely contains the true difference in sensitivity/specificity between SSC and DHS questionnaires. We used the two one-sided tests approach to calculate this interval [55],[56].

## Results

### Participants

**Figure 2:**
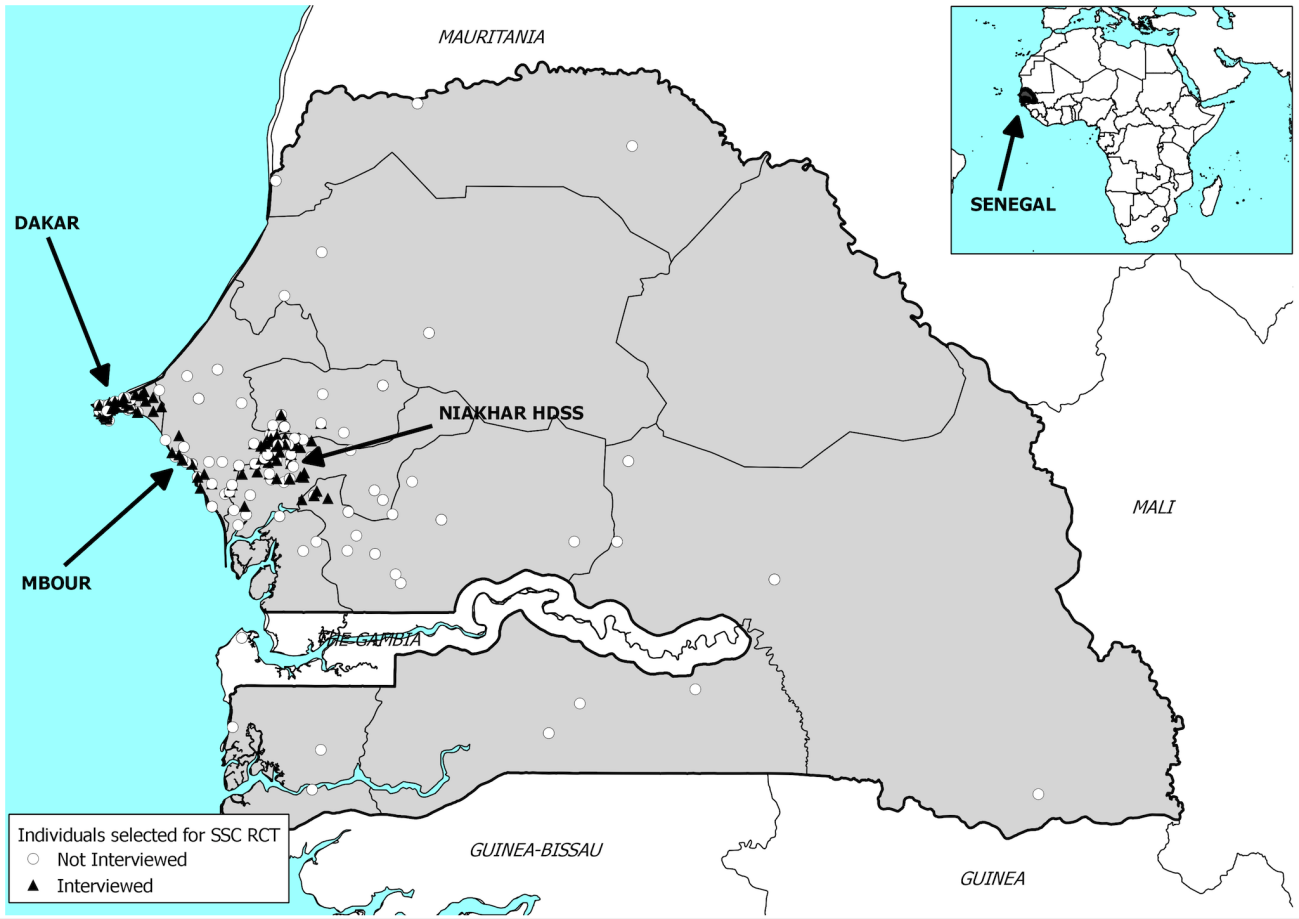
Map of the study site. Location of study participants in Senegal. Black triangles represent interviewed respondents, and white circles represent sampled individuals who did not participate in the validation study, either because they refused, were absent, or could not be traced. RCT, randomized controlled trial.

The study was conducted between 15 January 2013 and 27 March 2013. In total, 609 participants were assigned to complete a DHS questionnaire, and 580 a SSC questionnaire (Figure 1). On average, participants had approximately six known siblings according to the HDSS dataset (Table 1). The most recent adult death recorded by the HDSS among the siblings of a respondent occurred on average 10.0–10.7 y ago for the sisters of respondents and 11.7–11.9 y ago for their brothers. Among siblings who had died within 15 y of the surveys, the corresponding figures were 6.5–6.9 y for deceased sisters and 7.2–7.3 y for deceased brothers. The average age of participants was close to 34 y old. More than a third of the participants were interviewed outside of the HDSS area. The geographical distribution of study participants is shown in Figure 2. The average educational level was low, and the majority of participants were Muslim. For all these characteristics, there were no significant differences between respondents assigned to the SSC versus DHS group (Table 1).

**Table 1:** Characteristics of study participants, by study group.

Category	Characteristic	DHS Questionnaire		SSC Questionnaire		*p*-Value
		*N*	Mean or Proportion	*N*	Mean or Proportion	
**Sibship characteristics**	**Expected number of siblings**	609	6.4^a^	580	6.3^a^	0.62
	**Time since most recent adult death (years)**					
	Among all adult sisters of the respondent	128	10.0^b^	123	10.7^b^	0.47
	Among adult sisters who died in the past 15 y	99	6.5^b^	95	6.9^b^	0.52
	Among all adult brothers of the respondent	296	11.7^b^	288	11.9^b^	0.83
	Among adult brothers who died in the past 15 y	201	7.2^b^	200	7.3^b^	0.92
**Respondent characteristics**	**Age (years)**	609	33.3	580	34.2	0.19
	**Sex**					0.37
	Male	294	48.3%	295	50.9%	
	Female	315	51.7%	285	49.1%	
	**Place of interview**					0.81
	Niakhar HDSS area	375	61.6%	361	62.2%	
	Outside of Niakhar HDSS area	234	38.4%	219	37.8%	
	**Education**					0.94
	No schooling	349	57.7%	329	56.9%	
	Primary schooling	129	21.3%	123	21.3%	
	Secondary schooling or higher	127	21.0%	126	21.8%	
	**Religion**					0.07
	Muslim	487	80.4%	448	78.1%	
	Christian	115	19.0%	126	21.9%	
	Other	4	0.6%	—	—	
	**Ethnic group**					0.58
	Sereer	581	95.7%	554	96.8%	
	Wolof	8	1.3%	5	0.9%	
	Other	18	3.0%	13	2.3%	
	**Languages spoken**					
	Sereer	600	98.9%	574	99.3%	0.41
	Wolof	585	96.4%	561	97.1%	0.51

*p*-Values are based on a χ^2^ test of the difference between both groups of participants for categorical variables or a *t*-test for continuous variables.

a This is the mean number of expected siblings per respondent. Expected siblings are siblings ever registered by the Niakhar HDSS.

b This is the mean number of years since a respondent's adult sister/brother most recently died.

### Estimates

#### Effects of the SSC on data quality

In both questionnaires, the proportion of missing data on survival outcomes was low (Table S2). Only 0.07%–0.15% of reported siblings had missing data on vital status, 0.42%–0.56% of reported siblings had missing data on age at death, and 0.56%–0.90% of reported siblings had missing data on date of death. On all those variables, there were no significant differences in the likelihood of missing data between the DHS and SSC groups. The proportion of missing data on the current age of live siblings was significantly lower in the SSC group than in the DHS group: 0.53% of live siblings reported in the DHS group had missing data on age versus only 0.04% in the SSC group (*p*<0.01).

**Figure 3:**
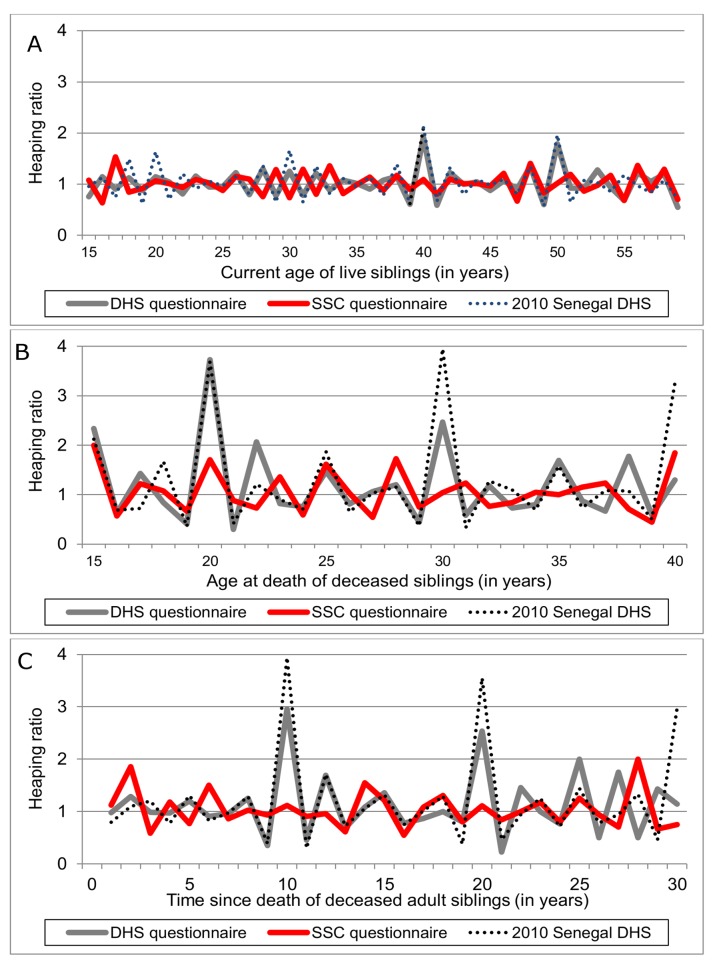
Age/date heaping in siblings' survival histories, by study group. (A) Current age of live siblings; (B) age of sibling at death; (C) date of sibling death. The time series represented here are heaping ratios calculated as indicated in Equation 1. In (B), we did not include data on deaths at ages 40 y and above because of limited sample size. Deaths at ages 40 y and above are, however, included in (C).

The three panels of Figure 3 report the extent of age and date heaping in the DHS and SSC questionnaires. Figure 3A shows notable heaping in reports of current age of live siblings. This occurs at ages 40 and 50 y in the DHS group, similar to what was observed during the 2010/2011 Senegal DHS (the Senegal DHS also shows additional heaping at ages 20 and 30 y). On the other hand, there is no apparent heaping of current age of live siblings in the SSC group. Figure 3B shows heaping of reported age of death among siblings deceased at an adult age. It shows significant heaping at ages 15, 20, 22, and 30 y in the DHS group. Heaping was particularly strong at age 20 y (ratio>3.5). Heaping of reported ages at death in the DHS group was similar to, although slightly less pronounced than, what was reported in the 2010/2011 Senegal DHS. On the other hand, even though there was some evidence of heaping at age 15 y in SSC group (ratio = 2), there was no heaping in the SSC at later ages of death. Finally, Figure 3C shows heaping in reports of date of death. In the DHS group, there was significant heaping 10, 20, and 25 y before the survey (again similar to what was observed in the 2010/2011 DHS). Such heaping was not present in the SSC group.

#### Effects of the SSC on data accuracy

The SSC had significantly higher sensitivity in recording adult female deaths than the DHS questionnaire (Table 2). Among respondents in sibships with at least one adult female death in the past 15 y according to the HDSS dataset, 75.6% reported a concordant SSH in the DHS group versus 89.6% in the SSC group (*p* = 0.027). We did not detect a significant difference in specificity between the DHS and SSC questionnaires in recording adult female deaths, but equivalence tests suggested that this difference was less than 5.5 percentage points. We did not detect a significant difference in the sensitivity of the DHS versus SSC questionnaires in recording adult deaths among the brothers of respondents. An equivalence test suggested that this difference was likely less than 7.0 percentage points. Finally, there were no significant differences in specificity between the SSC and DHS questionnaires in recording adult deaths among the brothers of a respondent, with equivalence tests suggesting that this difference was likely less than 4.0 percentage points.

**Table 2:** Specificity and sensitivity of SSH data, by study group.

Reporting of Deaths	Sensitivity^a^							Specificity^b^						
	DHS Group Concordant/Total	SSC Group Concordant/Total	Adjusted Analysis				Equivalence Δ^e^	DHS Group Concordant/Total	SSC Group Concordant/Total	Adjusted Analysis				Equivalence Δ^e^
			aOR^c^(95% CI)	DHS^d^	SSC^d^	*p*-Value				aOR^c^(95% CI)	DHS^d^	SSC^d^	*p*-Value	
Adult female deaths	75/99	85/95	2.83 (1.13, 7.07)	75.6	89.6	0.027	—	466/510	456/485	1.51 (0.87, 2.64)	91.3	94.0	0.146	5.5
Adult male deaths	173/201	170/200	0.94 (0.51, 1.73)	85.8	85.1	0.848	7.0	364/408	340/380	0.99 (0.58, 1.69)	89.4	89.3	0.973	4.0

Concordant SSH data are those in which a respondent reported a sibship composition that matches the sibship composition in the HDSS dataset (e.g., if an adult female death was recorded by the HDSS among the sibship, the respondent also reported such a death during the SSH data collection). “Total” is the total number of respondents used for each calculation.

a Sensitivity is defined as the proportion of concordant SSHs among respondents from sibships with at least one adult female/male death recorded by the HDSS.

b Specificity refers to the proportion of concordant SSHs among respondents from sibships without any adult female/male deaths recorded by the HDSS.

c Adjusted odds ratios (aORs) measure changes in the odds of a concordant answer associated with the use of the SSC; they are obtained from adjusted analyses using logistic regressions with stratifying variables as covariates.

d The values of sensitivity and specificity are predicted probabilities obtained from these logistic regressions; *p*-values are also obtained from these logistic regressions. Standard errors are adjusted for the clustering of respondents within sibships.

e Δ is the equivalence margin such that the absolute value of the difference in sensitivity/specificity between the DHS and SSC groups is |DHS−SSC|<Δ. It is obtained through a two one-sided tests approach following [56]. We arrived at the Δ recorded above through an iterative process: it is the smallest value of Δ for which both one-sided tests of the null hypothesis of no equivalence are rejected at the *p*<0.05 level. We did not calculate Δ for the difference in sensitivity between DHS and SSC for adult female deaths because the difference was significant at the *p*<0.05 level.

**Figure 4:**
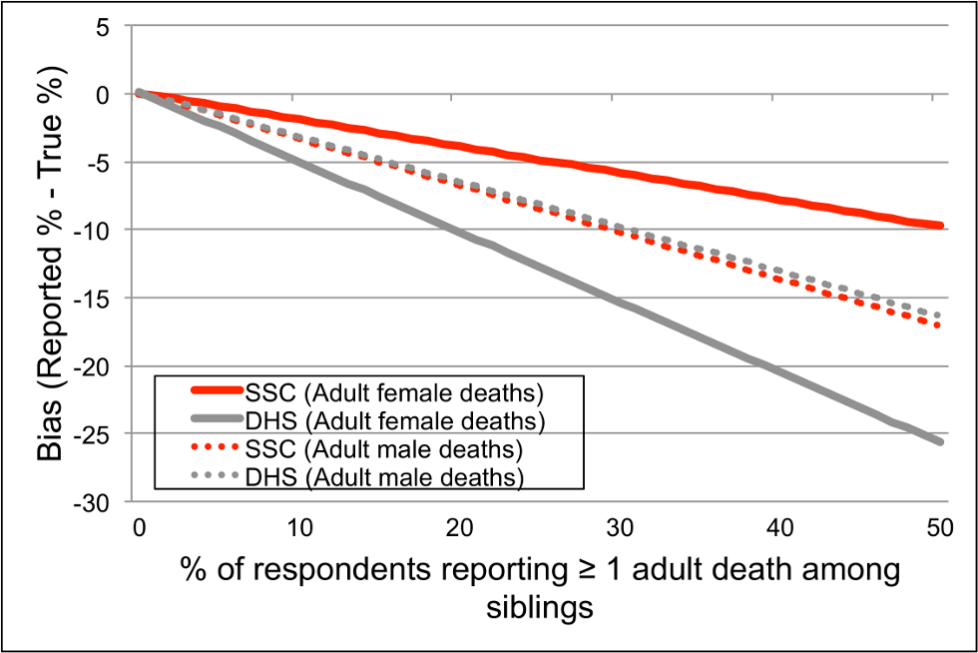
Effects of the SSC on bias in the proportion of respondents who report having had at least one death among their adult siblings. The true proportion of respondents with one or more deaths among their adult sisters or brothers was calculated using the values of sensitivity and specificity in Table 2, along with the formula for 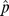 indicated in Equation 2 (derived in [50]).

In Figure 4, we show how the SSC affects estimates of the proportion of respondents who have experienced at least one adult death among their sisters/brothers. Both questionnaires underestimated this proportion. The extent of bias increased with the proportion of respondents who reported an adult death among their sisters/brothers. The SSC, however, reduced bias in reporting of adult female deaths. For example, in two similar SSC and DHS surveys during which 10% of respondents reported an adult death among their sisters, the calculated extent of bias was only 1.9 percentage points in the SSC survey versus 5.0 percentage points in the DHS survey. In settings where 30% of respondents reported an adult death among their sisters, the corresponding figures were 5.8 percentage points for the SSC versus 15.3 percentage points for the DHS questionnaire. There were no differences in the extent of bias in the reporting of adult male deaths.

#### Subgroup analyses

**Figure 5:**
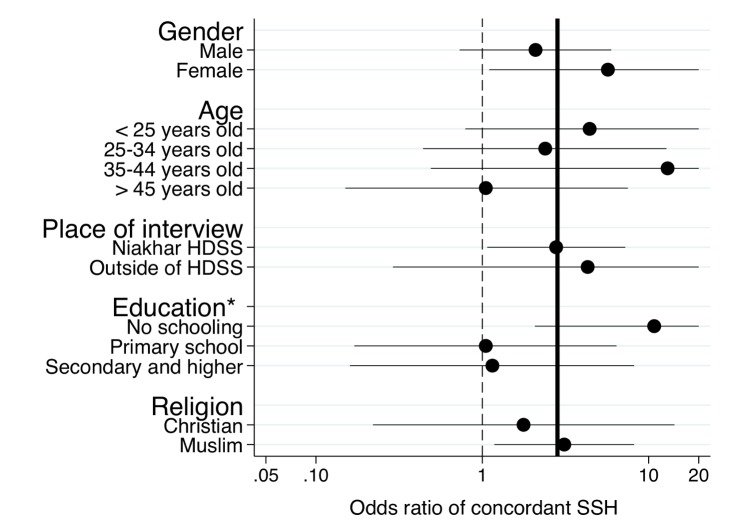
Subgroup analyses of the effects of the SSC on the sensitivity of SSH data for adult female deaths. The analytical sample for the analyses is constituted of respondents with at least one adult death among their sisters according to the HDSS dataset. The circles in the graphs represent odds ratios of reporting an adult female death associated with use of the SSC questionnaire among respondents in these sibships. Error bars represent 95% confidence intervals. Confidence intervals are clipped at 20. Estimates of odds ratios are obtained from stratified Mantel-Haenszel odds ratios. We tested for homogeneity of odds ratios across all strata of the classifying variable. The thick line represents estimates of the odds ratios for the full sample of the validation study. The dashed line corresponds to an odds ratio equal to 1. *p<0.1, tests of significance refer to the test of the homogeneity of the odds ratio across strata of the classifying variable.

In subgroup analyses (Figure 5), we found some evidence that the effects of the SSC on the reporting of adult female deaths varied by educational level of the respondent (*p* = 0.055, test of the homogeneity of odds ratios). The odds of reporting a concordant SSH associated with use of the SSC were higher among respondents who had never been to school than among other respondents. We found no evidence of heterogeneity in the effects of the SSC on the reporting of adult female deaths across other respondent characteristics.

**Figure 6:**
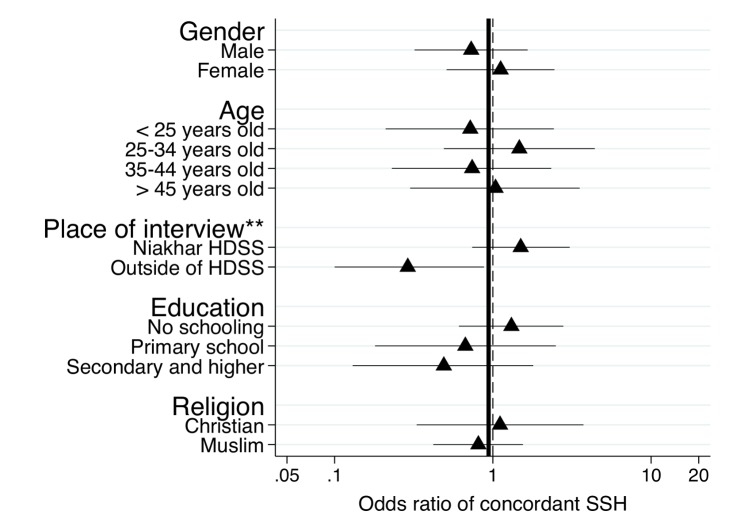
Subgroup analyses of the effects of the SSC on the sensitivity of SSH data for adult male deaths. The analytical sample for the analyses is constituted of respondents with at least one adult death among their brothers according to the HDSS dataset. The circles in the graphs represent odds ratios of reporting an adult male death associated with use of the SSC questionnaire. Error bars represent 95% confidence intervals. Estimates of odds ratios are obtained from stratified Mantel-Haenszel odds ratios. We tested for homogeneity of odds ratios across all strata of the classifying variable. The thick line represents estimates of the odds ratios for the full sample of the validation study. The dashed line corresponds to an odds ratio equal to 1. **p<0.05, tests of significance refer to the test of the homogeneity of the odds ratio across strata of the classifying variable.

In Figure 6, we found that the effects of the SSC on the reporting of adult male deaths varied by where the respondent was interviewed. The odds of reporting a concordant SSH associated with use of the SSC were significantly higher among respondents interviewed in the Niakhar HDSS area relative to respondents interviewed in migratory situations (test of homogeneity, *p* = 0.001). There was no heterogeneity in the effects of the SSC on the specificity of SSH data in recording both male and female adult deaths.

#### Effects of the SSC on inter-sibling reliability

Table 3 shows agreement in reporting of adult deaths among siblings of the same sibship, by study group. We found that inter-sibling agreement about the occurrence of adult female deaths in a respondent's sibship was significantly higher in the SSC group than in the DHS group (Kappa = 0.87 versus 0.61). There were no significant differences in inter-sibling agreement about adult male deaths between the DHS and SSC groups.

**Table 3:** Reliability of adult mortality data, by study group.

Type of Adult Death	Crude Agreement between Siblings			Kappa	
	DHS Group (Percent)	SSC Group (Percent)	*p*-Value	DHS Group	SSC Group
Adult female deaths in past 15 y	86.7	95.1	0.013	0.61	0.87
Adult male deaths in past 15 y	84.1	80.6	0.424	0.68	0.61

These calculations are limited to 295 sibships in which two siblings were interviewed during the validation study. *p*-Values are based on a χ^2^ test of the difference between both groups of the validation study; Kappa is calculated as follows:

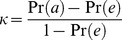
where a represents agreement between two siblings, and e is the hypothetical probability of chance agreement.

#### Differences in interview duration

**Figure 7:**
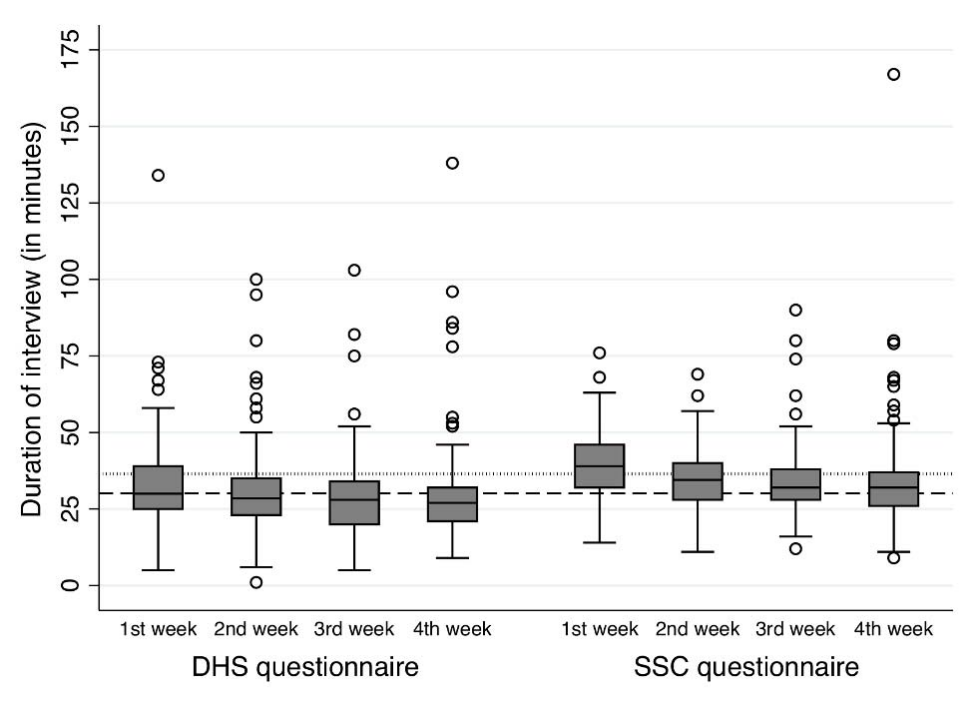
Distribution of interviewing time by study group and week of fieldwork. In this figure, the bottom and top of each box represents the first and third quartiles of the distribution of interviewing time, and the middle line represents the median. The whiskers represent the most extreme values within 1.5 times the interquartile range; circles represent outliers. Two interviews reported to have lasted 472 and 625 min are excluded from these figures for visualization purposes, but were included in calculations of median time. These interviews lasted exceptionally long because they were discontinued and the interviewer subsequently left the respondent's house at the time of interruption. The interviewer later returned to finish the interview, but did not record the time at which they discontinued the interview, nor did they record the time at which they resumed it. As a result, the actual interviewing time is not known. Five interviews reported to have lasted more than 100 min are included in this graph. These interviews were also momentarily interrupted, but the interviewer remained at the respondent's house until completing the interview. Data on interview duration were missing for 45 interviews. Of these, 24 were in the DHS group (3.9%) and 21 were in the SSC group (3.7%).

We compared the duration of interviews in the DHS versus SSC groups. These data were missing for 21 interviews in the SSC group (3.7%) versus 24 interviews in the DHS group (3.9%). The median duration of the entire interview (i.e., including also socio-demographic questions) was 30.1 min in the DHS group versus 36.5 min in the SSC group (*p*<0.001). The distribution of interview duration by study group is shown in Figure 7. In both study groups, the median duration of interview declined over time: for example, in the SSC group, during the first of week of fieldwork, interviews lasted a median of 39.4 min, but median duration declined to 34.5 min in week 4. In the DHS group, corresponding values were 32.6 min versus 29.4 min.

## Discussion

SSHs are rapidly becoming the primary source of data for adult mortality estimates in countries with limited vital registration [3],[18], but they are affected by (potentially large) reporting errors. We tested whether an enhanced SSH questionnaire (i.e., the SSC) improved the quality of adult mortality data collected during surveys. The key findings of this study were as follows: (1) the SSC significantly reduced age and date heaping in SSH data compared to the DHS questionnaire; (2) the SSC significantly improved the sensitivity of SSH data in recording adult female deaths; (3) the SSC did not improve the sensitivity of SSH data in recording adult male deaths; (4) the SSC did not reduce the specificity of SSH data in recording adult deaths either among men or women (i.e., it did not lead to additional reports of adult deaths in families where no such death had occurred); and (5) the SSC took a median 6 min more to complete than the DHS questionnaire (36.5 min versus 30.1 min). These results are consistent with those of several other studies that showed that the use of event history calendars reduces age/date heaping in demographic surveys [33],[36],[38],[39]. They are also consistent with prior trials of recall cues and supplementary interviewing techniques to improve the completeness of survey data on social relationships [27],[29].

### Why Did the SSC Improve the Reporting of Adult Deaths among Only the Sisters of a Respondent?

There are several reasons why the SSC may have improved the sensitivity of SSH data in recording adult deaths among the sisters of a respondent, but not among his/her brothers. First, the sensitivity of the DHS questionnaire (control group) was higher in recording adult male deaths than adult female deaths (85.8% versus 75.6%). There was thus less “room” for improving the reporting of adult male deaths using the SSC. The higher quality of SSH reporting about male deaths in the DHS questionnaire may be due to living arrangements of brothers and sisters in the Niakhar area. Among the Sereer of Senegal, marriage is patrilocal, i.e., women move into their husband's family compound after marriage, whereas men—even after marriage—remain with their kinship group. These residential arrangements imply that someone is separated from his/her sister(s) much more frequently, whereas s/he will often stay in close contact with his/her brothers. In this context, the recall cues we included may have been more effective at eliciting additional sisters than they were at eliciting additional brothers.

Second, the sample of adult female deaths included in this validation study was possibly more selective than the sample of adult male deaths. Indeed, a mother ID number (necessary to identify sibships to be included in the validation study) was available for only 45.9% of women who had died at an adult age within the past 15 y versus 66.6% of men who had died at an adult age within that time frame (Table S1). If the adult female deaths that could be included in the validation study were more responsive to recall cues than the included adult male deaths, then sample selectivity may explain the observed gender differential in the effects of the SSC.

Third, the lack of overall SSC effect on the reporting of adult male deaths is largely due to the lower sensitivity of the SSC in capturing adult deaths among the brothers of respondents interviewed in migratory situations (Figure 6). Among residents of the HDSS area, on the other hand, the SSC appeared slightly more sensitive than the DHS questionnaire in capturing adult deaths among respondents' brothers, although the difference was not statistically significant (Figure 6). There are several factors related to fieldwork organization that may have affected the reporting of adult deaths among respondents interviewed in migratory settings. If these factors disproportionately affected the reporting of the survival of a respondent's brother(s), then they could potentially explain why the SSC did not improve the reporting of adult male deaths. For example, SSC interviews with migrants were conducted last, after close to 2 mo of fieldwork. Interviewers may have experienced significant fatigue at that time, and may thus have been less likely to follow SSC instructions (e.g., sensitize respondents, use recall cues). Similarly, we collected the information necessary for migrant follow-up during our first passage in each study village (i.e., at the time of the DHS interviews). As a result, migrant follow-up was conducted in the DHS group within 3 wk of the collection of migration data versus >6 wk in the SSC group. This gap between collection of migration data and migrant follow-up may have led to errors in recruitment. This may have affected the composition of the pool of migrants we were able to trace for SSC interviews. For example, the proportion of migrants interviewed in Dakar was similar for both male and female respondents in the DHS group (62.1% versus 57.3%, *p* = 0.47) but not in the SSC group (64.3% versus 52.5%, *p* = 0.05). The allocation of interviewers to study areas (i.e., who conducted interviews in the Niakhar HDSS area versus migratory settings) also differed slightly between the DHS and SSC groups because of housing and transport availability, or personal leave. Interviewers less apt to elicit adult male deaths may thus have conducted more interviews with migrants in the SSC group.

Finally, a large number of interviews in migratory settings took place either late at night (i.e., after 9 pm) or at a respondent's place of employment. In these contexts, respondents may have been worried about the time it would take to complete the SSC. Interviewers, for example, reported that respondents in Dakar expressed concerns about the size of the “grid” (i.e., the A3 paper printout of the SSC). Concerns about interview duration may thus have prompted respondents to omit some of their siblings to complete the SSC interview process faster. Unfortunately, we have limited ways to test whether interviewer fatigue and allocation, as well as respondents' perceptions, affected the reporting of adult male and female deaths differently. Further research is thus needed to (1) understand the reasons for the less accurate reporting of adult male deaths among migrants in the SSC and (2) develop new supplementary interviewing techniques that may help address this issue.

### Study Limitations

Our work has several important limitations. First, the reference dataset (i.e., the Niakhar HDSS) we used to evaluate the accuracy of SSH data is not a gold standard. Data on the composition of sibships may be inaccurate, incomplete, or missing when sibship members were born outside of the HDSS area. Similarly, deaths occurring to sibship members who have migrated outside of the HDSS area (or who have never resided in the area) are not recorded by the HDSS. These limitations of the reference dataset imply that our estimates of the specificity and sensitivity of SSH data in recording adult deaths may sometimes be biased. For example, in sibships where the HDSS did not record any adult death, some adult deaths reported by respondents may have occurred outside of the HDSS area or may have been missed during the course of HDSS follow-up. The limitations of the reference dataset, however, affect both groups of the validation study in a similar manner and should not affect our assessment of the effectiveness of the SSC.

Second, the analyses we report here are unplanned exploratory analyses of the validation study data. As a result, they are more likely to yield false positive results (e.g., detecting a difference in sensitivity between SSC and DHS questionnaires when, in fact, such a difference does not exist). The likelihood of false positive results was also increased by the fact that we tested multiple hypotheses, e.g., we tested the effects of the SSC separately by gender of the reported sibling.

Third, the statistical power of some of the tests we conducted was limited. For example, we could not investigate heaping of ages of death above 40 y because of limited sample size (Figure 3). Similarly, some of the tests of differences in sensitivity/specificity by questionnaire type were based on small sample sizes (Table 2). We conducted equivalence tests to explore the likely magnitude of these differences, but some of the equivalence intervals we obtained were fairly wide and thus little informative (Table 2).

Fourth, since we started data collection with the DHS group and then continued with the SSC group using the same interviewers, improvements in data quality observed in the SSC group may have been due to general gains in interviewing skills during the course of the study (rather than to “true” SSC effects). We tried to control for such “training effects” or “experience effects” by design, by hiring experienced interviewers who had already collected SSH data during at least one the Senegal DHS. However, training effects cannot be ruled out entirely: interviewers may have taken some time reacquiring SSH interviewing skills during the first few days of fieldwork in the control group. Future validation studies should explore using study designs in which interviewers are randomly allocated to study groups (i.e., SSC versus DHS).

Finally, we could not investigate whether improvements in data quality/accuracy translate into more accurate estimates of adult mortality indicators such as _45_q_15_, i.e., the probability of dying between the ages of 15 and 59 y old. Indeed, it is not possible to directly compare the adult mortality rates obtained from different SSH questionnaires to the HDSS data because of the censoring of HDSS data for migrants (see above). In that context, comparing SSH and HDSS data requires identifying exactly which sibling reported during a SSH was lost to follow-up in the HDSS because of migration. Such record linkages [19] are necessary to make the HDSS and SSH datasets comparable by either excluding siblings lost to HDSS follow-up or imputing a censoring date in the SSH datasets.

Instead of this direct comparison of adult mortality indicators between SSH and HDSS data, we explored the potential impact of the SSC on estimates of the proportion of respondents with at least one adult death in their sibship. This parameter is an important component of adult mortality indicators. Indeed, the total number of deaths at ages 15–59 y old reported during a survey (denoted _45_ *D*_15_, i.e., the numerator of adult mortality rates) is the product of (1) the proportion of respondents who report at least one adult death in their sibship and (2) the average number of adult deaths reported per sibship. In Figure 4, we showed that, under observed levels of specificity and sensitivity (Table 2), both the SSC and the DHS questionnaires underestimate the proportion of respondents who have experienced at least one adult death among their sisters. Relative to the DHS questionnaire, however, the SSC significantly reduced bias in estimates of this proportion for the reporting of adult female deaths. The extent of bias reduction was especially large in settings where a high proportion of respondents reported having experienced an adult death among their sisters.

All else being equal, the SSC should thus reduce the extent of bias in estimates of _45_q_15_ for women. Further research on the impact of the SSC on estimates of adult mortality is, however, necessary. Such research will need to consider differences in (1) reports of the clustering of deaths within sibships (i.e., number of deaths per sibship) and (2) calculation of person-years lived by the siblings of a respondent (i.e., the denominator of adult mortality rates).

### External Validity of Study Results

There are also important questions regarding the external validity of our study results, i.e., whether we can expect the observed effectiveness of the SSC to extend to other populations and surveys in Senegal and elsewhere. The SSH data we collected in the DHS group during the validation study appeared comparable to the SSH data collected during the 2010/2011 Senegal DHS: in particular, age and date heaping patterns were largely similar in the two datasets (Figure 3). This similarity suggests that the results of our assessment of the SSC may also extent to other settings, including national surveys.

There are, however, several potential threats to external validity. First, we could only trace migrants who had moved to regions of Senegal that were the closest to Niakhar or Dakar (see Figure 2). These migrants likely maintained more ties to their siblings who still resided in the HDSS area than other migrants who moved to less accessible parts of the country. This latter group would, however, be included in national surveys. If the quality and accuracy of retrospective SSH reports depends on the frequency of interaction between siblings, then the extent of reporting errors in SSH data may be higher in national studies than what we observed during this validation study. Second, because of repeated interactions with HDSS interviewers during household visits, residents of the Niakhar HDSS may also be more aware of ages and dates of vital events than other populations. If that is the case, then the quality of SSH data collected during the validation study is likely significantly higher than the quality of data collected in national surveys such as DHS. Third, the data collection protocol we followed differed from the protocol used in DHS and other nationally representative surveys. In particular, during DHS, SSH data are collected after several additional modules on reproductive and child health, sexual behaviors, etc., so that both interviewer and respondent fatigue may be significantly higher than during our validation study. Finally, even though we hired former DHS interviewers, we could not install the same supervisory structure, and quality control procedures, as in national DHS.

### Extensions

Future research on the SSC and other SSH data quality improvement approaches should extend in several directions. First, micro-simulations should be used to explore the potential impact of the SSC on (1) adult mortality levels and trends (see above) and (2) estimated gender and socioeconomic differentials in adult mortality. Since the SSC improves data quality particularly for women, it has the potential to modify our assessment of gender differences in adult mortality, including the contribution of pregnancy-related deaths to mortality differentials. Similarly, the SSC appeared to improve the reporting of adult female deaths particularly among the least educated respondents (Figure 5). Respondents without formal education may indeed be less familiar with ages and dates than other respondents and may experience more difficulties in completing the DHS questionnaire. These respondents may thus have benefited from the use of the event history calendar in the SSC to more accurately date the deaths that had affected their siblings. These socioeconomic differentials in reporting behaviors have potentially important implications for estimates of the burden of disease. If specific health conditions disproportionately affect the least educated (e.g., pregnancy-related deaths, chronic diseases), then using the SSC may lead to a better appreciation of the extent of health inequalities in a population.

Second, the SSC itself should be refined in multiple ways. New recall cues and probing strategies could be added that target siblings who are still omitted by respondents or that stimulate respondents who are more likely to report incomplete SSHs (e.g., migrants). The SSC should be adapted for use on tablets and other mobile data collection supports. Most DHS (and other nationally representative surveys) are now conducted on such supports. Using mobile data collection tools could further increase the ease of SSC data collection. It may also help integrate new probes and improve compliance with SSC instructions, and it may alleviate respondents' concerns about the length of the SSH interview.

Third, further validation studies of the SSC should be conducted in diverse epidemiological, societal, and data collection settings. For example, future SSC tests should include populations of southern Africa that are affected by large HIV epidemics, as well as matrilineal societies, in which men move into their wife's family compound (and thus are separated from their own siblings) after marriage. The effects of the SSC on SSH data quality may be different in such settings. Eventually, tests of the SSC should be nested within national SSH surveys to ascertain whether this data collection tool improves national estimates of adult mortality.

In summary, this work suggests a new approach to improving estimates of adult mortality in countries with limited vital registration. Instead of relying solely on analytical procedures to correct reporting errors, demographers should also seek to improve the quality of the input data used in burden of disease models and mortality estimates. We showed here that this can be achieved using simple interviewing tools that have been used successfully in a number of fields of demographic and epidemiological research.

## Supporting Information

Table S1 **Availability of mother ID number among population members ever registered by the Niakhar HDSS, by vital status.**
(DOCX)

Table S2 **Missing data in siblings' survival histories, by study group.**
(DOCX)

Alternative Language Abstract S1 **French translation of the abstract by BM, GP, and SH.**
(DOCX)

Checklist S1 **STARD checklist for validation study.**
(DOC)

Checklist S2 **CONSORT checklist for randomization of questionnaire allocation and data analysis.**
(DOC)

Questionnaire S1 **Copy of the siblings' survival calendar used during the validation study (in French).**
(PDF)

Questionnaire S2 **Copy of the migration inquiry form used to collect contact information about absent residents and migrants (in French).**
(DOC)

Text S1 **Study protocol.**
(DOCX)

## Abbreviations

CSS: corrected sibling survival
DHS: Demographic and Health Surveys
HDSS: Health and Demographic Surveillance System
SSC: siblings' survival calendar
SSH: siblings' survival history
