# Supplementary material for: Improving the Quality of Adult Mortality Data Collected in Demographic Surveys: Validation Study of a New Siblings' Survival Questionnaire in Niakhar, Senegal
Source: PLoS Med. 2014 May 27;11(5):e1001652. doi: 10.1371/journal.pmed.1001652 (PMC4035258; doi:10.1371/journal.pmed.1001652)
Supplement: Table S1 — Availability of mother ID number among population members ever registered by the Niakhar HDSS, by vital status. (DOCX) [file pmed.1001652.s001.docx]

|  | **Alive at last HDSS round** | | | **Lost to HDSS follow-up** | | | **Deceased before last HDSS round** | | | | | |
| --- | --- | --- | --- | --- | --- | --- | --- | --- | --- | --- | --- | --- |
|  |  |  |  |  |  |  | **Within past 15 years** | | | **More than 15 years ago** | | |
|  | Available | Unavailable | p-value | Available | Unavailable | p-value | Available | Unavailable | p-value | Available | Unavailable | p-value |
| **Gender** |  |  | 0.000^1^ |  |  | 0.003^1^ |  |  | 0.000^1^ |  |  | 0.000^1^ |
| **Male** | 11,171 (97.4) | 291  (2.6) |  | 4,843 (63.8) | 2,749  (36.2) |  | 371  (66.6) | 189  (33.4) |  | 232  (33.5) | 461  (66.5) |  |
| **Female** | 11,059  (95.3) | 518  (4.7) |  | 7,739 (65.9) | 4,012  (34.1) |  | 226  (45.9) | 266  (54.1) |  | 81  (12.0) | 597  (88.0) |  |
| **Age** |  |  | 0.000^2^ |  |  | 0.000^2^ |  |  | 0.000^2^ |  |  | 0.000^2^ |
| **Current Age/age at exit/age at death^3^** | 30.1 (11.8) | 44.0  (11.2) |  | 14.8 (10.7) | 20.5  (9.6) |  | 35.0  (13.3) | 42.8  (11.9) |  | 28.3  (10.9) | 41.9  (12.7) |  |
| **Date** |  |  | -- |  |  | 0.000^2^ |  |  | 0.000^2^ |  |  | 0.000^2^ |
| **Time since exit/death** | -- | -- |  | 17.9  (11.0) | 19.6  (8.9) |  | 6.6  (4.2) | 10.8  (3.2) |  | 26.3  (8.7) | 28.4  (9.4) |  |

**Table S1: availability of Mother ID number among population members ever registered by the Niakhar HDSS, by vital status**

*Notes: ^1^* p-value is based on a χ^2^ test of the association between two categorical variables. ^2^ based on a t-test. ^3^ calculations limited to adults aged 15-59 years old; for those lost to follow-up, we limited our calculations to those who would have been 15-59 at the time of the survey
