# Supplementary material for: Improving the Quality of Adult Mortality Data Collected in Demographic Surveys: Validation Study of a New Siblings' Survival Questionnaire in Niakhar, Senegal
Source: PLoS Med. 2014 May 27;11(5):e1001652. doi: 10.1371/journal.pmed.1001652 (PMC4035258; doi:10.1371/journal.pmed.1001652)
Supplement: Table S2 — Missing data in siblings' survival histories, by study group. (DOCX) [file pmed.1001652.s002.docx]

|  | DHS questionnaire | | SSC questionnaire | | p-value |
| --- | --- | --- | --- | --- | --- |
|  | N | % missing | N | % missing |  |
| Vital status | 4,125 | 0.07 | 3,900 | 0.15 | 0.278 |
| Current age (live siblings) | 2,464 | 0.53 | 2,290 | 0.04 | 0.002 |
| Age at death (deceased siblings) | 1,658 | 0.42 | 1,604 | 0.56 | 0.570 |
| Time since death (deceased siblings) | 1,658 | 0.90 | 1,604 | 0.56 | 0.251 |

**Table S2: missing data in siblings’ survival histories, by study groups**

*Notes:* p-values are based on a χ^2^ test of the difference between both groups of the validation study; they are adjusted for the clustering of siblings’ reports within respondents, and the clustering of respondents within sibships.

.
