# Supplementary material for: Improving the Quality of Adult Mortality Data Collected in Demographic Surveys: Validation Study of a New Siblings' Survival Questionnaire in Niakhar, Senegal
Source: PLoS Med. 2014 May 27;11(5):e1001652. doi: 10.1371/journal.pmed.1001652 (PMC4035258; doi:10.1371/journal.pmed.1001652)
Supplement: Alternative Language Abstract S1 — French translation of the abstract by BM, GP, and SH. (DOCX) [file pmed.1001652.s003.docx]

Résumé en français

Contexte
Dans les pays où les décès ne sont pas tous déclarés à l’état civil, la mortalité des adultes est estimée habituellement en utilisant des informations sur l’histoire des frères et sœurs et de leur survie (notées SSH pour « siblings’ survival histories ») recueillies par les enquêtes démographiques et de santé (EDS). Les déclarations des enquêtés sur leurs frères et sœurs sont affectées par des erreurs de déclaration. Nous avons développé un nouveau questionnaire pour recueillir les SSH, appelé le « calendrier de la survie des frères et  »sœurs" (et noté SSC pour « siblings’ survival calendar »). Il intègre des questions supplémentaires pour limiter les omissions de frères et sœurs et utilise un calendrier d’histoire de vie pour améliorer le recueil des dates et des âges. Nous avons émis l'hypothèse que le SSC permettait d'améliorer la qualité des données sur la mortalité des adultes.

Méthodes et résultats
Nous avons mené une étude de validation rétrospective parmi la population du système de surveillance démographique de Niakhar  au Sénégal. Nous avons interrogé des hommes et des femmes de 15-59 ans avec soit le questionnaire EDS, soit le SSC (la répartition a été faite au hasard). Nous avons comparé les déclarations recueillies dans chaque groupe avec les données du suivi démographique de Niakhar. Le SSC réduit la tendance des répondants à arrondir les dates et les âges au plus proche multiple de cinq ou de dix ("l’attraction pour les chiffres ronds "). Le SSC enregistre mieux aussi les décès de femmes adultes (sa sensibilité est meilleure) : parmi les répondants dont au moins une sœur est décédée à l’âge adulte au cours des 15 dernières années, 89,6 % ont déclaré un décès de femme adulte au cours des interviews menés avec le SSC contre 75,6 % dans les entretiens menés avec le questionnaire EDS (p = 0,027). La spécificité du SSC est similaire à celle du questionnaire EDS, c'est à dire que le nouveau questionnaire n'a pas augmenté le nombre de déclarations de décès erronées. Cependant, le SSC n'a pas amélioré la déclaration des décès qui concernent les frères des enquêtés. L’étude a plusieurs limites dont la sélectivité de l'échantillon, une faible validité externe, et une multiplicité des tests statistiques.

Conclusions
Le SSC permet de recueillir des informations sur l’histoire de la fratrie plus précises que le questionnaire utilisé dans les EDS. Des recherches complémentaires sont nécessaires pour évaluer les effets du SSC sur les estimations des taux de mortalité chez les adultes. Des études de validation supplémentaires devraient être menées dans différents contextes sociaux et épidémiologiques.
