# Supplementary material for: Improving the Quality of Adult Mortality Data Collected in Demographic Surveys: Validation Study of a New Siblings' Survival Questionnaire in Niakhar, Senegal
Source: PLoS Med. 2014 May 27;11(5):e1001652. doi: 10.1371/journal.pmed.1001652 (PMC4035258; doi:10.1371/journal.pmed.1001652)
Supplement: Questionnaire S1 — Copy of the siblings' survival calendar used during the validation study (in French). (PDF) [file pmed.1001652.s006.pdf]

IDENTIFICATION

VILLE/VILLAGE: ____________________________ HAMEAU/QUARTIER: ____________________________

CHEF DE CONCESSION____ |__|__|__|__|__|__|__|__|

ENQUETE(E): ____ |__|__|__|__|__|__|__|__|

CONJOINT: ____ |__|__|__|__|__|__|__|__|

PERE:_____ ____ |__|__|__|__|__|__|__|__|

MERE:_____ ____ |__|__|__|__|__|__|__|__|

VISITE	DATE	ENQUETEUR	RESULTAT*	LANGUE**	RDV	SAISIE
1						
2						
3						

* CODE RESULTAT : 1 = Rempli, 2 = Pas a la maison, 3 = différé, 4 = Refus, 5 = Partiel, 6 = Incapacité, 7 = autre (préciser)
** CODE LANGUE: 1 = Serer, 2 = Wolof, 3 = autre (préciser); cela fait référence a la langue de l'entretien, pas a la langue du questionnaire.

CARACTERISTIQUES SOCIODEMOGRAPHIQUES DE L'ENQUETE(E)

101. HEURE DE DEBUT: |___|___| : |___|___|

102.	En quels mois et année êtes-vous ne(e)?<br>*"98" si connait pas le mois, "9998" pour l'année*	MOIS: |___|___| ANNEE: |___|___|___|___|
103.	Quel age aviez-vous a votre dernier anniversaire?	AGE EN ANNEES REVOLUES: |___|___|
104.	Etes-vous allé(e) a l'école?	OUI 1<br>NON 2 (→ 106)
105.	Quel est le plus haut niveau d'études que vous avez atteint?	ELEMENTAIRE 1<br>MOYEN 2 (→ 111)<br>SECONDAIRE 3 (→ 111)<br>SUPERIEUR 4 (→ 111)<br>AUTRE(____________________) 5 (→ 111)
106.	Avez-vous déjà participé a un programme d'alphabétisation ou a un autre programme qui comprenait l'apprentissage de la lecture et de l'écriture (primaire non compris)?	OUI 1<br>NON 2 (→ 111)
108.	Dans quelles langues étaient donnes les programmes d'alphabétisation auxquels vous avez participes?<br>*Enregistrez tout ce qui est mentionne.*	ARABE/MEDERSA A<br>WOLOF B<br>SERER C<br>AUTRE(________________________________) D
111.	Quelle est votre religion?	MUSULMAN 1<br>CHRETIEN 2<br>ANIMISTE 3<br>SANS RELIGION 4<br>AUTRE(________________________________) 5
112.	Quelle est votre ethnie?	SERER 1<br>WOLOF 2<br>AUTRE(________________________________) 5
113.	Est-ce que vous parlez suffisamment bien les langues suivantes?	**SERER** 1 = OUI 2 = NON<br>**WOLOF** 1 = OUI 2 = NON<br>**FRANCAIS** 1 = OUI 2 = NON
114.	Quand avez-vous eu une conversation en__________ pour la dernière fois?<br>*(1 = aujourd'hui, 2 = dans la semaine, 3 = > 1 semaine, 4 = ne sait plus, 98 = jamais)*	**SERER** 1 2 3 4 98<br>**WOLOF** 1 2 3 4 98<br>**FRANCAIS** 1 2 3 4 98

CALENDRIER DES FRERES ET DES SŒURS
INSTRUCTIONS POUR L'ENQUETEUR

**1ERE ETAPE : CALENDRIER DE L'ENQUETE(E) (P.4)**	
1. Introduire le calendrier a l'enquêté(e) en utilisant la formulation suivante : « serigne (soxna ci) doma bougon niou waxtan ci xew xew yi gana feuss si sa doundou wala sa diar-diar mo xam. Figa moussa deuk, - seuil, wala diang ».	|_________|
2. Amorcer le calendrier de l'enquêté(e) en inscrivant dans le champ "naissance(s)" la date de naissance de l'enquêté(e) obtenue en Q102 et/ou Q103.	|_________|
3. Aborder les différents champs avec l'enquêté(e) en commençant par les évènements	|_________|
**2EME ETAPE : LISTE DES FRERES ET SOEURS (P.3)**	
1. Obtenir la liste des frères et sœurs en suivant les procédures	|_________|
**3EME ETAPE : CALENDRIER DES FRERES ET SOEURS (P.5)**	
1. Ordonner la liste des frères et sœurs selon le rang de naissance	|_________|
2. Inscrire les prénoms et obtenir le sexe de tous les frères et sœurs	|_________|
3. Obtenir des informations sur les âges, la survie et les dates de décès des frères et sœurs.	|_________|
4. Calculer l'âge au décès de chaque frère et sœur (NA, si le frère ou la sœur sont toujours en vie)	|_________|
5. Vérifier les intervalles de naissance entre les frères et sœurs, relancer sur les intervalles longs (> 4 ans)	|_________|

LISTE DES FRERES ET SŒURS DE L'ENQUETE(E)

Maintenant, je voudrais vous poser des questions sur vos frères et sœurs, c'est-à-dire sur tous les enfants nés de votre *mère biologique.* Pourriez-vous me donner la liste de tous vos frères et soeurs de même mère biologique? Dans l'ordre ou ils vous viennent a l'esprit.

*FS1 :*______________________________
*FS2 :*______________________________
*FS3 :*______________________________
*FS4 :*______________________________
*FS5 :*______________________________
*FS6 :*______________________________
*FS7 :*______________________________
FS8 : ______________________________
FS9 : ______________________________
*FS10 :*______________________________
FS11 : ______________________________
FS12 : ______________________________
FS13 : ______________________________
*FS14 :*______________________________

Relire la liste en partant du dernier frère/soeur mentionné(e) puis lui demander: " y'a-t-il d'autres frères et/ou soeurs que vous n'avez pas mentionnés ?"

*NS1:*______________________________
*NS2:*______________________________
*NS3:*______________________________
*NS4:*______________________________

Aide-mémoire: Au cours d'enquêtes précédentes, nous nous sommes rendus compte que beaucoup de gens oublient de mentionner certains de leurs frères et soeurs, même s'ils ont l'impression d'avoir mentionné tout le monde. Les questions que je vais vous poser maintenant visent à vous aider à vous rappeler de vos frères et sœurs que vous avez peut-être oublié de me déclarer.

1. Résidence: J'ai ici une liste d'endroits où les gens de ce village migrent fréquemment, soit parce qu'ils y travaillent, ou parce qu'ils se sont mariés là-bas. Est-ce que certains de vos frères et sœurs, **que vous n'avez pas mentionnes jusqu'à présent**, ont migré vers ces endroits? 1 = Niakhar, 2 = Ngoye/Bambeye, 3 = le diakhao, 4 = Fatick, 5 = Mbour, 6 = Dakar et sa region, 7 = Thies, 8 = Kaolack, 9 = Koupentoum, 10 = Casamance, 11 = ailleurs.

*MG1:*______________________________
*MG2:*______________________________
*MG3:*______________________________
*MG4:*______________________________

2. Statut vital: parfois, on peut aussi oublier de mentionner une frère ou une soeur car il/elle est décédé. Je sais qu'il peut être douloureux de se souvenir du décès d'un proche, mais est-ce qu'il y a des frères/sœurs, décédés **que vous n'avez pas mentionnés jusqu'à présent**?

*SV1:*______________________________
*SV2:*______________________________
*SV3:*______________________________
*SV4:*______________________________

3. Proximité: parfois, on peut aussi oublier de mentionner un frère ou une soeur car on les a peu connus ou on les côtoie peu souvent. Par exemple, peut-être avez-vous des frères/soeurs qui sont nés bien avant ou après vous et qui n'ont pas vécu longtemps avec vous? Ou d'autres avec qui vous avez peu de relations?

*PR1:*______________________________
*PR2:*______________________________
*PR3:*______________________________
*PR4:*______________________________

4. Père biologique: **4. Père biologique:** enfin, on peut aussi oublier de mentionner un frère ou soeur car ils sont issus d'une autre union, c'est-à-dire qu'ils ont la même mère biologique, mais pas le même père biologique que nous. Est-ce qu'il y a des frères et sœurs, d'un autre père biologique, **que vous n'avez pas mentionnés jusqu'à présent ?**

*PB1:*______________________________
*PB2:*______________________________
*PB3:*______________________________
*PB4:*______________________________

Pour récapituler, en tout, votre mère a eu ___ enfants, dont vous.

ANNEE	DUREE	EVENEMENTS NATIONAUX OU PERSONNELS	ENQUETE(E)			
			NAISSANCE(S)<br>*Enquete(e) = N*<br>Enfants de l'enq, =prenom	UNION(S)<br>*DV = divorce*<br>*SP= separation*<br>*V = veuvage*	RESIDENCE	ECOLE
1953	60					
1954	59					
1955	58					
1956	57					
1957	56					
1958	55					
1959	54					
1960	53	Indépendance				
1961	52					
1962	51					
1963	50					
1964	49					
1965	48					
1966	47					
1967	46					
1968	45	Grève étud.				
1969	44	Décès M'BACKE				
1970	43					
1971	42					
1972	41					
1973	40	Sécheresse				
1974	39	Création PDS				
1975	38					
1976	37					
1977	36					
1978	35	SENGHOR réélu				
1979	34					
1980	33					
1981	32	A. DIOUF prés.				
1982	31					
1983	30	A. DIOUF élu				
1984	29					
1985	28					
1986	27					
1987	26					
1988	25	Année blanche				
1989	24	Evèn. Maur-SN				
1990	23					
1991	22	Décès A.LAHAD				
1992	21	Acc. SONACOS				
1993	20	Elections				
1994	19					
1995	18					
1996	17					
1997	16	Décès DABAKH				
1998	15					
1999	14					
2000	13	A.WADE élu				
2001	12	11 SEPT				
2002	11	Joola				
2003	10					
2004	9	Deces Card. Thiandoum				
2005	8					
2006	7					
2007	6	WADE réélu				
2008	5	B. OBAMA élu				
2009	4	Décès S.SALIOU				
2010	3	Décès Y.K SENE				
2011	2					
2012	1	M. SALL élu				
2013	0					

ANNEE	DUREE	FRERES & SŒURS								
		#1	#2	#3	#4	#5	#6	#7	#8	#9
		M F NSP	M F NSP	M F NSP	M F NSP	M F NSP	M F NSP	M F NSP	M F NSP	M F NSP
Avt.	>									
1953	*60*									
1954	*59*									
1955	*58*									
1956	*57*									
1957	*56*									
1958	*55*									
1959	*54*									
1960	*53*									
1961	*52*									
1962	*51*									
1963	*50*									
1964	*49*									
1965	*48*									
1966	*47*									
1967	*46*									
1968	*45*									
1969	*44*									
1970	*43*									
1971	*42*									
1972	*41*									
1973	*40*									
1974	*39*									
1975	*38*									
1976	*37*									
1977	*36*									
1978	*35*									
1979	*34*									
1980	*33*									
1981	*32*									
1982	*31*									
1983	*30*									
1984	*29*									
1985	*28*									
1986	*27*									
1987	*26*									
1988	*25*									
1989	*24*									
1990	*23*									
1991	*22*									
1992	*21*									
1993	*20*									
1994	*19*									
1995	*18*									
1996	*17*									
1997	*16*									
1998	*15*									
1999	*14*									
2000	*13*									
2001	*12*									
2002	*11*									
2003	*10*									
2004	*9*									
2005	*8*									
2006	*7*									
2007	*6*									
2008	*5*									
2009	*4*									
2010	*3*									
2011	*2*									
2012	*1*									
2013	*0*									
Age (DC)		|__|__|	|__|__|	|__|__|	|__|__|	|__|__|	|__|__|	|__|__|	|__|__|	|__|__|

ENQUETEUR : IDENTIFIER LES DÉCÈS AYANT EU LIEU A UN ÂGE > A 12 ANS PUIS INSCRIRE LES NOMS ET NUMÉROS DES PERSONNES DÉCÉDÉ(E)S. ENFIN POSER LES QUESTIONS SUIVANTES.

	**Prénom/Nom**	________<br>________	________<br>________	________<br>________	________<br>________	________<br>________
	**Numéro**	|___|	|___|	|___|	|___|	|___|
	**Sexe**	**M F NSP**	**M F NSP**	**M F NSP**	**M F NSP**	**M F NSP**
!!! NOTE POUR L'ENQUETEUR, LES QUESTIONS 210-213 CI-DESSOUS NE SONT À POSER QUE POUR LES FEMMES DECEDEES. POUR UN DECES D'HOMME, PASSER AUX QUESTIONS 214 IMMEDIATEMENT.						
210	Est-ce que (NOM) était enceinte au moment du décès ?	1 = oui (→ 213)<br>2 = non	1 = oui (→ 213)<br>2 = non	1 = oui (→ 213)<br>2 = non	1 = oui (→ 213)<br>2 = non	1 = oui (→ 213)<br>2 = non
211	Est-ce que (NOM) est décédée au cours d'un accouchement ?	1 = oui (→ 213)<br>2 = non	1 = oui (→ 213)<br>2 = non	1 = oui (→ 213)<br>2 = non	1 = oui (→ 213)<br>2 = non	1 = oui (→ 213)<br>2 = non
212a	Est-ce que (NOM) est décédée dans les **42 jours** suivant un la fin d'une grossesse ou d'un accouchement ?	1 = oui (→ 213)<br>2 = non	1 = oui (→ 213)<br>2 = non	1 = oui (→ 213)<br>2 = non	1 = oui (→ 213)<br>2 = non	1 = oui (→ 213)<br>2 = non
212b	Est-ce que (NOM) est décédée dans les **12 mois** suivant la fin d'une grossesse ou d'un accouchement ?	1 = oui<br>2 = non	1 = oui<br>2 = non	1 = oui<br>2 = non	1 = oui<br>2 = non	1 = oui<br>2 = non
213	A combien d'enfants vivants est-ce que (NOM) a donné naissance au cours de sa vie?	|___|___|	|___|___|	|___|___|	|___|___|	|___|___|
!!! NOTE POUR L'ENQUETEUR : LES QUESTIONS 214A, 214B, 214C CI-DESSOUS SONT A POSER A PROPOS DE TOUS LES DECES A AGE > 12 ANS. PAS SEULEMENT LES DECES DE FEMMES						
214b	Est-ce que (NOM) est décédé(e) suite a une blessure ?	1 = oui<br>2 = non	1 = oui<br>2 = non	1 = oui<br>2 = non	1 = oui<br>2 = non	1 = oui<br>2 = non
214c	Etait-ce du a un(e) :<br>*Si suicide, passer au décès suivant*	Accident 1<br>Suicide 2<br>Homicide 3<br>Guerre 4<br>Cata. Nature 5	Accident 1<br>Suicide 2<br>Homicide 3<br>Guerre 4<br>Cata. Naturelle 5	Accident 1<br>Suicide 2<br>Homicide 3<br>Guerre 4<br>Cata. Nature 5	Accident 1<br>Suicide 2<br>Homicide 3<br>Guerre 4<br>Cata. Nature 5	Accident 1<br>Suicide 2<br>Homicide 3<br>Guerre 4<br>Cata. Nature 5
214d	Donner le détail des événements qui ont conduit a la blessure ?	Véhicule à moteur 1<br>Accident piéton/vehic. 2<br>Moto 3<br>Vélo 4<br>Chute 5<br>Arme à feu 6<br>Mine / bombe 7<br>Arme blanche 8<br>Incendie 9<br>Poison 10<br>noyade 11<br>Autre blessure 12	Véhicule à moteur 1<br>Accident piéton/vehic. 2<br>Moto 3<br>Vélo 4<br>Chute 5<br>Arme à feu 6<br>Mine / bombe 7<br>Arme blanche 8<br>Incendie 9<br>Poison 10<br>noyade 11<br>Autre blessure 12	Véhicule à moteur 1<br>Accident piéton/vehic. 2<br>Moto 3<br>Vélo 4<br>Chute 5<br>Arme à feu 6<br>Mine / bombe 7<br>Arme blanche 8<br>Incendie 9<br>Poison 10<br>noyade 11<br>Autre blessure 12	Véhicule à moteur 1<br>Accident piéton/vehic. 2<br>Moto 3<br>Vélo 4<br>Chute 5<br>Arme à feu 6<br>Mine / bombe 7<br>Arme blanche 8<br>Incendie 9<br>Poison 10<br>noyade 11<br>Autre blessure 12	Véhicule à moteur 1<br>Accident piéton/vehic. 2<br>Moto 3<br>Vélo 4<br>Chute 5<br>Arme à feu 6<br>Mine / bombe 7<br>Arme blanche 8<br>Incendie 9<br>Poison 10<br>noyade 11<br>Autre blessure 12

Pour chaque frère et sœur cite dans le calendrier, obtenir des informations sur la résidence des frères et des sœurs au moment de l'enquête (frères/sœurs vivants) ou au moment du décès:

Frere/sœur	*Num*	*Village/ville*	*Chef de concession*	*Autre nom ? surnom ?*	*Nom du Mari/ de la femme*	*Même père biologique ?*
	1					1 = oui<br>2 = non
	2					1 = oui<br>2 = non
	3					1 = oui<br>2 = non
	4					1 = oui<br>2 = non
	5					1 = oui<br>2 = non
	6					1 = oui<br>2 = non
	7					1 = oui<br>2 = non
	8					1 = oui<br>2 = non
	9					1 = oui<br>2 = non
	10					1 = oui<br>2 = non
	11					1 = oui<br>2 = non
	12					1 = oui<br>2 = non
	13					1 = oui<br>2 = non
	14					1 = oui<br>2 = non
	15					1 = oui<br>2 = non

Conditions de l'entretien :

Langue préférée ?	*A apporte des réponses?*	*A traduit des questions ?*	*Heure de fin*
1 = Serer<br>*2 = wolof*<br>*3 = autre*<br>________________	*1 = frère/sœur (même mère)*<br>*2 = frère/sœur (≠ mère)*<br>*3 = enfant*<br>*4 = conjoint*<br>*5 = autre : ______________________*<br>*6 = aucun*	*1 = frère/sœur (même mère)*<br>*2 = frère/sœur (≠ mère)*<br>*3 = enfant*<br>*4 = conjoint*<br>*5 = autre :_____________________*<br>*6 = aucun*	|___|___| : |___|___|

Remarques :

____
____
____
____
____
____
____
____
____
____
____
____
____
____
