# Supplementary material for: Improving the Quality of Adult Mortality Data Collected in Demographic Surveys: Validation Study of a New Siblings' Survival Questionnaire in Niakhar, Senegal
Source: PLoS Med. 2014 May 27;11(5):e1001652. doi: 10.1371/journal.pmed.1001652 (PMC4035258; doi:10.1371/journal.pmed.1001652)
Supplement: Questionnaire S2 — Copy of the migration inquiry form used to collect contact information about absent residents and migrants (in French). (DOC) [file pmed.1001652.s007.doc]

# ENQUÊTE SUR LA MORTALITE ADULTE A NIAKHAR (Janvier – Mars 2012)

# Fiche migration

| 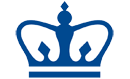 | 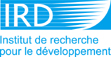 | 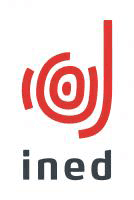 |
| --- | --- | --- |
| Columbia UniversityNew York - USA | Institut de recherche pour le développement (IRD)Dakar - Sénégal | Institut national d’études démographiques (INED)Paris - France |
| **IDENTIFICATION** | | |
| ENQUETE(E) : |___||___||___||___||___|Nom de l’enquêté(e) : …………………………….…….………………Village/ville : |___||___||___| Nom du village/ville : ………………………………………………….Hameau/quartier : |___||___||___| Nom du Hameau/quartier : …………………………………………..*Concession*: |___||___||___| Nom du chef de *concession* : …………………………………………..Conjoint : |___||___||___||___||___| Nom du conjoint : ………………………………………….……….Père : |___||___||___||___||___| Nom du père : ………………………………………………….. Mère : |___||___||___||___||___| Nom de la mère : ….………………………………………………. | | |
| **NOUVELLE ADRESSE/ NOUVEAU CONJOINT (si différent)** | | |
| Nom du village/ville : ……………………………………………………………………………………………Nom du Hameau/quartier ……………………………………………..…………………………………………….Nom du chef de *concession* : ………….…………………………………………………………………………Prénom et Nom du conjoint : ……………………………………………………………………………….………….Prénom et Nom du père du conjoint : .………………………………………………………………………… Prénom et Nom de la mère du conjoint : …………………………………….…………………………………….  Numéro de téléphone : ………………………….…………………………………………………………………..  Autre information/contact permettant d’identifier l’enquêté(e) : ………………………………………………………..  ………………………………………………………..……………………………………………………………………….  ………………………………………………………..……………………………………………………………………….  ………………………………………………………..……………………………………………………………………….  ………………………………………………………..……………………………………………………………………….  Informateur/trice : ……….………………………………………………………………………….………….  Lien de parenté avec l’enquêté(e) : ….…………………………………………………………….………….  Concession : ………………………………………………………………………………………….…………. | | |
