# Supplementary material for: Improving the Quality of Adult Mortality Data Collected in Demographic Surveys: Validation Study of a New Siblings' Survival Questionnaire in Niakhar, Senegal
Source: PLoS Med. 2014 May 27;11(5):e1001652. doi: 10.1371/journal.pmed.1001652 (PMC4035258; doi:10.1371/journal.pmed.1001652)
Supplement: Text S1 — Study protocol. (DOCX) [file pmed.1001652.s008.docx]

1. Study purpose

The main aim of this study is to improve the measurement of adult mortality rates and causes in developing countries with limited vital registration systems. We propose to test whether a new survey instrument can improve the quality of adult mortality data collected during household-based surveys in developing countries. This instrument incorporates techniques proven to enhance recall of other topics and relations (e.g., dietary intake), but not currently used to collect adult mortality data. This project builds on a pilot study we previously conducted under protocol E9043.

1. Study rationale

With child mortality declining, an increasing proportion of all deaths in the developing world will occur at adult ages in the next decades. Adult mortality rates are high because of the impact of the AIDS epidemics, other infectious diseases (e.g., TB and other respiratory infections), reproductive conditions (e.g., maternal mortality, unsafe abortions), injuries and deaths due to external causes (e.g., suicide, homicide, road traffic), non-communicable diseases that affect primarily adults (e.g., cardiovascular diseases or neoplasms) and war deaths in crisis settings. Mortality at adult ages often has far-reaching implications because it affects the most economically active age groups. In resource-poor settings, adult deaths threaten the livelihood of entire families, prevent the schooling and development of children, limit support to the elderly, and precipitate economic hardship. To alleviate this impact, large amounts of resources are invested each year by national governments (in particular the US government) and international organizations in interventions aimed at preventing premature deaths or treating adult illnesses in developing countries, such as scaling-up HAART for AIDS treatment, DOTS for TB control and treatment, improving access to emergency obstetric care services, or preventing unsafe abortions. The ultimate impact of these interventions remains largely unknown however because existing estimates of adult mortality rates are frequently contested.

How is adult mortality measured in developing countries? The key indicator of adult mortality is 45q15, the life-table probability of dying between the ages of 15 and 59. Few developing countries have functioning vital registration systems to measure 45q15 accurately. Instead, estimates of adult mortality are extrapolated from child mortality indices and model life tables or are based on information on a respondent's close relatives collected during surveys and censuses. These include questions on the number of deaths in a respondent's household over the previous 12 months, the survival of a respondent's spouse (widowhood method), or parents (orphanhood method). Increasingly 45q15 is estimated using siblings' survival histories (SSH): respondents are asked to list all siblings born to their biological mother by birth order, and then to provide information about their gender, survival status, and current age; or age at death and time elapsed since death if the sibling is deceased. With SSH, adult mortality rates can be estimated directly

because both events and exposure time are known for each reported sibling.

What are the main barriers to accurate estimation of adult mortality in developing countries? Survey and census data on adult mortality in developing countries are becoming increasingly available. Measuring adult mortality through surveys and censuses however presents important challenges. Retrospective mortality data are affected by well-known sample selection biases. Individuals from high-mortality families are less likely to be interviewed in a survey, whereas individuals from healthy families may appear several times in a sample. Statistical procedures that effectively correct for sampling selection biases are now available and increasingly applied. On the other hand, retrospective survey data are affected by large recall biases. Respondents may forget to report some of their siblings, and may disproportionately forget to report deceased siblings during SSH interviews. In some settings, they may also include individuals that do not fit the survey definition of a sibling and leave out others that should have been included. Even if they report all of their siblings, they may misreport their siblings' ages.

How will the project improve our knowledge of adult mortality in developing countries? The consensus among demographers is that estimates of adult mortality in developing countries based on SSH under-estimate the true level of 45q15 because deaths are under-reported. Following the refinement of analytical techniques, this is now primarily due to forgetting of siblings during surveys and possibly interviewer behaviors. The proposed project seeks to develop a new strategy to elicit higher-quality siblings' survival histories. This strategy, the "siblings enhanced life calendar" (SELC), is based on i) cues designed to stimulate the recall of frequently omitted siblings and ii) a life history calendar approach to ascertaining the ages of siblings and the dates of events having affected them. We will test whether the SELC significantly reduces the proportion of siblings omitted during a survey. If that is the case, the proposed SELC will constitute a new approach to eliciting retrospective mortality data that can be tested on a larger scale and possibly incorporated in national surveys of adult mortality (e.g., DHS). It can also be extended to other types of demographic data on close relatives of a respondent including widowhood/orphanhood data. If the SELC does not prove effective in eliciting more complete reports of sibships, the proposed project will nonetheless have measured key, previously unobserved parameters such as the proportion of siblings forgotten during surveys and differences in age between reported/omitted siblings. Such parameters can be used to adjust estimates of 45q15.

Our approach to validating the SELC also improves on existing strategies to validate new survey instruments or new analytical techniques in demography. These have generally been conducted without a gold standard, by 1) gauging whether new estimates are consistent with other data sources at the aggregate level, or 2) testing whether the new instrument increases the reporting of a type of relations or behaviors assumed to be under-reported. Instead, we test, during a randomized controlled trial, whether SELC data reduces forgetting of siblings during surveys by linking individual survey reports of each sibling to a unique "gold standard" list of a respondent's siblings. This gold standard list has been constituted prospectively through demographic surveillance and in-depth genealogical surveys conducted in population observatories. Population observatories are small, fully enumerated and well-delimited populations in developing countries, which host a demographic surveillance system. By "demographic surveillance", we refer to the continuous monitoring of pregnancies, births, deaths, migrations and marriages during household visits (every 4-6 months). Demographic surveillance systems (DSS) provide the highest quality data on age and dates of demographic events in developing countries with no vital registration. Not all DSS however provide gold standard data against which to validate SSH data. This requires long time series of observations so that exhaustive lists of an individual's siblings can be constructed prospectively by linking each newborn to its mother over many years. Most DSS have been started in the last 10 years and thus do not allow that. Senegal however is home to one of the oldest population observatories: demographic surveillance in Niakhar began between 1962 and 1982. In addition, innovative procedures to accurately measure the age of population members prior to initiating demographic surveillance were implemented in each site. Detailed genealogies of each population member were collected from multiple sources prior to the start of demographic surveillance. These techniques allow ascertaining with confidence the age and the siblings of individuals born before the beginning of demographic surveillance. As a result, they provide unique "gold standard" data on the composition of sibships of respondents aged 15-59 in 2013.

1. Study design and statistical procedures

We test whether SELC reduces recall errors through a randomized controlled trial during which respondents will be randomized to completing a SSH interview using either the SELC (treatment arm) or the standard SSH instrument (control arm). The interview will be limited to the collection of adult mortality data and will not include other information elicited during national surveys of health (e.g., child health).

Study outcomes: The main outcome evaluated during this RCT is the proportion of respondents who forget at least one of their adult siblings during the survey. Secondary outcomes include the average error in reports of the i) current age of live siblings, ii) age at death of deceased siblings, iii) number of years since the death of deceased siblings.

Sample size determination: based on pilot data, we hypothesize that 14% of respondents forget at least one adult sibling during standard SSH (comparison group). We seek to detect small effects of the new strategy on this proportion. In order to detect a 50% decrease in that proportion (from 14 to 7.0%,), we will need to enroll 297 respondents in each study arm. Assuming a 85% participation rate, we will need to contact 349 respondents in each arm to reach that target. In total, the RCT sample size is 698 individuals.

Randomization procedures: First, a sample of 698 participants will be randomly drawn from the list of individuals in the population rosters of the Niakhar DSS who meet eligibility criteria (see below). Among these selected participants, randomization will be stratified by gender and age of the respondent (less than 25 / 25-44/ 45 and above). It will be done in blocks of 10 with computer-generated random numbers. This procedure ensures that the distribution of gender and age will be balanced across treatment and control groups.

Recruitment of interviewers/interviewer effects: Interviewers will be recruited among interviewers having participated in the 2010 Senegal DHS. To avoid interviewer effects, the same interviewers will conduct interviews in the treatment and in the control group. RCT Fieldwork will proceed as follows: 1st phase: control group data collection; 2nd phase: training in SELC interviews; 3rd phase: SELC data collection

Results from the RCT will be analyzed using simple two-sample statistical tests. Categorical outcomes (e.g., proportion of respondents with at least one forgotten sibling) will be compared using chi-square tests. Continuous outcomes (e.g., errors in reported ages or dates of deaths, average difference between reported and observed ages of a respondent's siblings) will be analyzed using t-tests or non-parametric tests if the errors in reporting of ages and dates are found to be non-normally distributed. We will test whether the use of the SELC reduces interviewer behavior biases by comparing the average age of forgotten siblings in the control and in the SELC arm. If the SELC prevent interviewers from skipping some of the respondent's siblings, then there should be no differences in age between forgotten and included siblings in the SELC arm.

1. Study procedures
2. Development of recall cues for the SELC

Recall cues are statements made before/during a survey interview to direct respondents to recall relations or events in which they are(were)involved, but may have forgotten. We develop recall cues by identifying which characteristics of siblings and of the sibling/respondent relationship make a sibling more likely to be forgotten during an interview. We will ask a random sample of 25 adult residents of the Niakhar DSS area to participate in a free and directed recall exercise (FDRE). The Niakhar population was chosen for convenience because it is the most easily accessible. The FDRE will proceed in 3 steps. First, respondents will be asked to list all the (maternal) siblings they can remember in the order that they come to their memory (free recall). Second, study interviewers will ask them to order the recalled siblings by birth order. Interviewers will document the potential difficulties respondents encounter in ordering their siblings by age. Finally, respondents will be presented with the list of their known siblings derived from demographic surveillance. They will first be asked whether there is someone they don't recognize on that list then to rank all the known siblings on the list according to: 1) the frequency of their physical (e.g., visits) and non-physical (e.g., phone calls, letters) interactions, 2) the strength of their relationship (e.g., very close/somewhat close/not close at all), and 3) the date of their last encounter with each sibling. They will also be asked how frequently they send/receive money to/from a particular sibling, whether they help/receive help from that sibling for agricultural or fishing activities, domestic chores (e.g., cooking). Study interviewers will carefully record the order in which respondents in the FDRE freely recall their siblings. We hypothesize that characteristics of a respondent-sibling relationship constitute key cognitive structures respondents use to recall their siblings during SSH. Analysis of the FDRE data will entail assessing whether the order in which siblings are freely recalled by respondents depends on some of the other rankings and categorizations of siblings elicited during the FDRE (e.g., frequency of interaction). We will do so using rank-order correlations.

1. Identification of adult deaths

Adult deaths in the study population are identified through demographic surveillance. Demographic surveillance sites (DSS) routinely collect data on vital events (births, deaths, migration, marriages and pregnancies) in small delimited (often rural) populations. All vital demographic events occurring within DSS populations are updated through regular visits by fieldworkers to each household, in general every year. Thus, during these visits, new events such as births, deaths, marriages, in and out migration and obvious pregnancies are registered. The dates of such events are also recorded. Pregnancies are monitored during these quarterly visits until they are terminated. This is to help improve on birth and death reporting, by capturing neonatal deaths in particular. For every vital event that is recorded, detailed information is collected using appropriate event registration forms. Genealogies and/or sibships are reconstructed by collecting data on biological parents. This intensive monitoring process has yielded high quality data on the population dynamics of rural sub-Saharan populations for which such data were previously unavailable.

1. Identification of respondents

The initial census of the study population included a genealogical survey, to collect genealogies going up to known ascendants and then down to living collateral relatives. One use of the genealogies in the project is to get detailed information on the relationships between the members of a compound and in particular the relationship of each one with the head of the compound. It also allows identifying with high precision the biological siblings of a population member. Each individual in the DSS databases is attributed a Mother ID #, which solely identifies the biological mother of a population member. Two individuals with the same mother ID # are thus maternal siblings. Very few individuals included in the DSS database are missing their mother ID #. It may happen if:

- Their mother was dead at the time of first enumeration or at the time of birth registration

- Their mother was residing elsewhere at the time of first enumeration or at the time of birth registration

- The information on the identification and residence of their mother provided during household visits was not specific enough to identify the mother in the household rosters.

As a result, sisters from the same biological mother may occasionally not be identified as such if one of the siblings falls within these categories.

1. Tracing of migrant sisters of deceased individuals

The DSS databases also include detailed information on the destination and contacts of out-migrants: district, village and compound of destination, possibly contact number, and spouse's name if migration is due to marriage. As a result, it is possible to trace and enroll migrant sisters of deceased women who currently reside outside of the DSS zone. This population is particularly important because recall of sibling survival may be affected by separation and distance. The quality of survey data could be particularly low among migrants who have been living away from their siblings for extended periods of time.

We plan to visit all such sisters who have migrated out of the zone and now reside in the departments of Fatick and Diourbel. We will also seek to enroll sisters having migrated to the greater Dakar region, where XX% of the population of Senegal resides. For convenience and due to budget constraints, we will not attempt to trace sisters having migrated to other parts of Senegal.

Tracing of migrant sisters will proceed as follows: the study team will use contact information (e.g., address, name of compound, possibly phone number) collected by the DSS to conduct an initial tracing. If the information provided does not allow locating the migrant, the study team will conduct further inquiries with relatives or co-residents living at the new address. Once in contact with the migrant, the study team will first administer a series of "filter" questions (based on DSS information) to ensure that the person contacted is indeed the sister of the deceased.

Such questions include place and date of birth, name of spouse (if married) and level of education. After these steps have been completed, migrant sisters will be interviewed according to the same protocol as the one used for sisters residing in the DSS area.

1. Study drugs and devices

n/a

1. Study questionnaires

The study will employ a different questionnaire in each study arm. In the control arm, study questionnaires are standard forms designed to record sibling survival histories, and used broadly during the demographic and health surveys in numerous sub-Saharan countries. In the treatment, we use the newly designed SELC. The SELC is a calendar indexed by year, which will stretch from 1930 to the year of the survey. 1930 is chosen because someone born in that year will reach age 60 in 1990 and we will seek to test the validity of the SELC for adult deaths having taken place within 20-25 years before the survey. The SELC's design will follow that of other studies of demographic events while incorporating several key innovations. It will be a large fold-out grid with units of time in years noted across the top of the grid. A preliminary, truncated sketch of the SELC to be used is attached. National and local "landmark" events will be obtained from local informants. The top portion of the grid ("respondent") records information on the respondent's life course, with each domain ("birth", "location" etc) providing timing cues supposed to help ascertain the sequencing and dates of events affecting siblings. The set of personal domains to be ascertained tentatively includes a respondent's own residence, schooling, marriage and childbearing histories. The bottom portion ("sibling #n") is used to record events in the history of a sibling. The same information set as in the closed-ended standard SSH questionnaire is elicited. Age of the sibling however is calculated indirectly, by computing the duration between two dates: birth and the date of the survey (or death). Sets of standard probes to ensure consistency and improve recall of siblings will be developed with fieldworkers and supervisors. Such probes will be based on i) recognition of gaps and long intervals in the respondent's mother's birth history (e.g., 3+ years between births of siblings may indicate

omission in the Senegalese populations), ii) inquiries about co-residing household members for each new residence of the respondent, and iii) other personal events/anecdotes that may be related by respondents during administration of the SHC. Siblings will be listed in the SELC in alphabetical order of the first name rather than by birth order: this will help prevent systematic under-reporting of younger siblings because of deliberate omission by interviewers of the siblings listed last in the interviewing sequence.

1. Study subjects

FDRE: Study subjects for the FDRE will be randomly selected residents of the Niakhar area aged 18-59. In total, we will conduct 25 FDRE interviews.

RCT of the new questionnaire (SELC): The RCT will be conducted among population members of Niakhar aged 15 to 59 . In total, 698 individuals will be contacted for enrollment in the study. Study participants will be selected at random among lists of residents of the study populations maintained through demographic surveillance.

The inclusion criteria are: to be aged 15-59 and to have at least one sibling ever registered by the DSS. This latter inclusion criteria is necessary because, for some members of the DSS population, there are no known siblings. As a result, the data they report during adult mortality surveys cannot be validated against DSS data. We will also oversample adults who have at least one sibling who has recently (i.e., since 1998) died at adult ages. This over-sample is necessary because we seek to test whether the new survey instrument prevents respondents from forgetting to report the death of their deceased siblings during surveys. Such forgetting of deceased siblings is indeed responsible for under-estimates of adult mortality. Because the proportion of adults aged 15-59 with a sibling who died at adult ages is low in each study population (<20%), we need to over-sample individuals who meet that criterion to guarantee statistical power during the RCT at a reasonable cost. Exclusion criteria for the SELC trial include: being outside of the age range and having participated in the FDRE in Niakhar. We expect that participants in the study will be composed of men and women in the same proportions as in the study populations.

Study participants will potentially include pregnant women if they are randomly selected among the list of population members meeting the inclusion criteria. Because the study is a RCT, study subjects will be randomly allocated to conducting the interview using the standard form of adult mortality questionnaire or the newly developed calendar form (SELC). Study participants will be asked to complete one single interview about their personal characteristics and the number and survival of their maternal siblings. It is expected that interviews will take ½ hour. There will be no planned follow-up.

1. Study recruitment

FDRE:

Study subjects for the FDRE exercise will be identified through the rosters of population members compiled and kept up-to-date by the demographic surveillance system of Niakhar. Recruitment will be solely through household visits. Unlike for the main trial (see below), there will be no attempt to trace migrants for participation in the FDRE.

RCT of the new questionnaire (SELC):

Study subjects will be initially identified through the rosters of population members compiled and kept up-to-date by the demographic surveillance system in Niakhar. These datasets permit identifying the subset of population who meet the eligibility criteria laid out above. They also include the last known residence of any population member (which village, which house within the village did the member reside in the last time s/he was seen by a demographic surveillance interviewer). Once the subset of eligible population members has been identified, we will sample 698 population members for inclusion in the proposed trial. Sampled respondents will be recruited into the study solely through household visits. They will be approached by study interviewers and will be offered participation in the study upon successful contact (after informed consent, see below). If a sampled population member has moved from his last known residence, interviewers will then inquire with other household members where s/he may have moved. If a population member has moved within the departments of Fatick orDiourbel or to the greater Dakar region, interviewers will elicit details of the new residence of that migrant as well as contact numbers (e.g., mobile phone number, or number of friend/relative who may be able to locate the migrant). Migrants will subsequently be contacted by the study team through household visits similar to the visits used for non-migrants. Each sampled population members will be visited by the study team up to a maximum of 3 times in case of absence or other temporary unavailability. There will be no fliers, billboards, radio messages or other similar recruitment procedures used during the study.

Informed consent for both the FDRE and the RCT of the new questionnaire will be elicited from sampled respondents prior to participation in each component (please that FDRE participants cannot participate in the RCT). Consent will be sought by study interviewers. Consent will be administered in the local languages by interviewers who are fluent in these languages. During the consent process, interviewers will provide key information on the research to the potential participants. This will include an indication that the study involves research, that participation is entirely voluntary and that they can refuse to participate, that they can discontinue the interview at any point in time without losing any benefits. The study interviewer will then describe the study procedures including how long the interview is going to last, what kind of questions will be asked. The study interviewer will clearly state that the study participant has the possibility to refuse to answer any single question s/he is not comfortable to answer. The reasons for inclusion, benefits, risks, discomforts and other information about the study necessary to make an informed decision about participation will be discussed with the participant. Written consent will be sought from the study participants unless they are illiterate; in that case, a thumbprint will be obtained and another adult household member will serve as witness.

Parental permission will be sought prior to the interview for individuals aged 15-17 years old who are not married. In Niakhar, women may marry prior to age 18; in this case, parental permission will not be sought and the woman herself will be asked to provide consent for study participation. Individuals aged 15-17 years old who are not yet married will be asked to provide assent. Parental permission and individual assent of participants aged 15-17 years old will be documented on the same form.

1. Confidentiality of study data

The data collected during this validation study are stored under a unique ID number attributed by DSS study teams to inhabitants of the study population. Access to identifying information about study participants is limited to study investigators. Data management and other tasks relating to study information are conducted solely on password-protected machines, and will take place in a limited-access computer center at the Institut de recherche sur le developement, in Dakar.

Data analysis will be conducted both in Dakar and at Columbia. Prior to data analysis, the datasets will be stripped of any identifying information (e.g., names, village number). New individual ID numbers will be attributed, which are not linked to any village and thus do not permit identification of the study respondent and his/her sibling(s). Transport of the study dataset from Dakar to Columbia will be on a portable hard drive. We chose this solution to avoid exposing the dataset to potential breach of confidentiality during transport through the internet. At Columbia, the analysis dataset will be stored on a password-protected network drive.

1. Potential risks

The study involves minimal risks. Risks associated with the study only involve possible discomforts felt by respondents when recalling deaths of siblings, as well as a potential breach of confidentiality if information collected during the study is accidentally disclosed to a third party. Procedures in place to minimize the risk of confidentiality breaches are described above.

1. Potential benefits

The study involves minimal risks. Risks associated with the study only involve possible discomforts felt by respondents when recalling deaths of siblings, as well as a potential breach of confidentiality if information collected during the study is accidentally disclosed to a third party. Procedures in place to minimize the risk of confidentiality breaches are described above.

1. Alternatives

The alternative to participating in the survey is simply not to participate.
